# Nutritional stress-induced regulation of microtubule organization and mRNP transport by HDAC1 controlled α-tubulin acetylation

**DOI:** 10.1038/s42003-023-05138-w

**Published:** 2023-07-25

**Authors:** Frank Wippich, Vaishali, Marco L. Hennrich, Anne Ephrussi

**Affiliations:** 1 Developmental Biology Unit, European Molecular Biology Laboratory (EMBL) Heidelberg, Meyerhofstrasse 1, Heidelberg, 69117 Germany; 2 Structural and Computational Biology Unit, European Molecular Biology Laboratory (EMBL) Heidelberg, Meyerhofstrasse 1, Heidelberg, 69117 Germany; 3 Present Address: Cellzome GmbH, GlaxoSmithKline, Heidelberg, Germany; 4 Present Address: Department of Cell and Developmental Biology, University College London, Gower Street, London, WC1E 6BT UK

**Keywords:** Oogenesis, Cytoskeleton, RNA transport

## Abstract

In response to nutritional stress, microtubules in cells of the *Drosophila* female germline are depleted from the cytoplasm and accumulate cortically. This triggers aggregation of mRNPs into large processing bodies (P-bodies) and oogenesis arrest. Here, we show that hyperacetylation of α-tubulin at lysine 40 (K40) alters microtubule dynamics and P-body formation. We found that depletion of histone deacetylase 1 (HDAC1) by RNAi phenocopies the nutritional stress response, causing α-tubulin hyperacetylation and accumulation of maternally deposited mRNPs in P-bodies. Through in vitro and in vivo studies, we identify HDAC1 as a direct regulator of α-tubulin K40 acetylation status. In well-fed flies, HDAC1 maintains low levels of α-tubulin acetylation, enabling the microtubule dynamics required for mRNP transport. Using quantitative phosphoproteomics we identify nutritional stress-induced changes in protein phosphorylation that act upstream of α-tubulin acetylation, including phosphorylation of HDAC1 at S391, which reduces its ability to deacetylate α-tubulin. These results reveal that *Drosophila* HDAC1 senses and relays the nutritional status, which regulates germline development through modulation of cytoskeleton dynamics.

## Abstract (web summary)

Quantitative phosphoproteomics shows that HDAC1 is hyperphosphorylated on S391 upon nutritional stress and analysis reveals phosphorylation of S391 plays a role in regulating deacetylase activity of HDAC1 towards α-tubulin and microtubule dynamics.

## Introduction

*Drosophila* oogenesis is tightly regulated by nutrient availability, which is sensed by the mother and transmitted to the developing germline cells via insulin signaling^1^. Upon nutrient deprivation, oogenesis arrests and large mRNP assemblies form in the oocyte and the interconnected nurse cells^1,2^. These membraneless assemblies have been called P-bodies based on their compositional similarity with their mammalian counterparts, which were shown to be sites of mRNA post-transcriptional control^3^. P-body formation in the fly germline depends in part on loss of intact microtubules, as microtubule depolymerizing drugs, such as colchicine, induce formation of similar RNP assemblies^2^, although the exact mechanism remains unclear.  A systemic insulin signal has been shown to activate insulin/TOR signaling in follicle cells, which regulates both microtubule organization and P-body formation in the underlying germline cells. Thus, reduced insulin signaling in nutrient-deprived conditions leads to cytoplasmic depletion and cortical condensation of microtubules^1^. However, neither the receiving nor the processing factors in the germline target cells have been identified thus far.

Post-translational modifications of α-tubulin are involved in microtubule dynamics and stability^4^. Acetylation of α-tubulin at K40, carried out primarily by α-tubulin acetyltransferase 1 (αTAT1), is known to alter microtubule dynamics by enhancing the flexibility of microtubules, thereby protecting them from mechanical stresses and making them more long-lived^5–7^. So far, changes in α-tubulin acetylation have been shown to affect multiple cellular processes, including intracellular trafficking^8^, and nutrient deprivation has been shown to induce hyperacetylation of α-tubulin^9^.

Histone deacetylases (HDACs), have been shown to regulate mRNP dynamics during stress and to coordinate stress granule formation via microtubule-based transport mechanisms in mammalian cells^10^. Moreover, cytoplasmic HDACs, most prominently class II HDACs, such as cytoplasmic HDAC6, can deacetylate α-tubulin in vitro and in vivo^11–13^.

Here, we identified deacetylation of α-tubulin on K40 by *Drosophila* HDAC1 as a regulatory step in the nutritional stress response in the female germline. We found that depletion of HDAC1 by RNAi phenocopies the nutritional stress response, causing hyperacetylation of α-tubulin, as well as accumulation of maternally deposited mRNPs in P-bodies. HDACs are themselves regulated by post-translational modifications, such as phosphorylation, to either modulate enzymatic activity, dimerization or subcellular localization^14–16^. Using quantitative phosphoproteomics of well-fed and nutritionally deprived egg chambers, we show that HDAC1 is hyperphosphorylated on S391 upon nutritional stress. We further demonstrate that phosphorylation of S391 plays a role in regulating the deacetylase activity of HDAC1, specifically toward α-tubulin. Taken together, these data indicate that HDAC1 is required to transmit nutritional information by modulating microtubule dynamics and microtubule-based mRNA transport in the *Drosophila* female germline.

## Results

### α-Tubulin acetylation affects oocyte mRNA transport

**Fig. 1:**
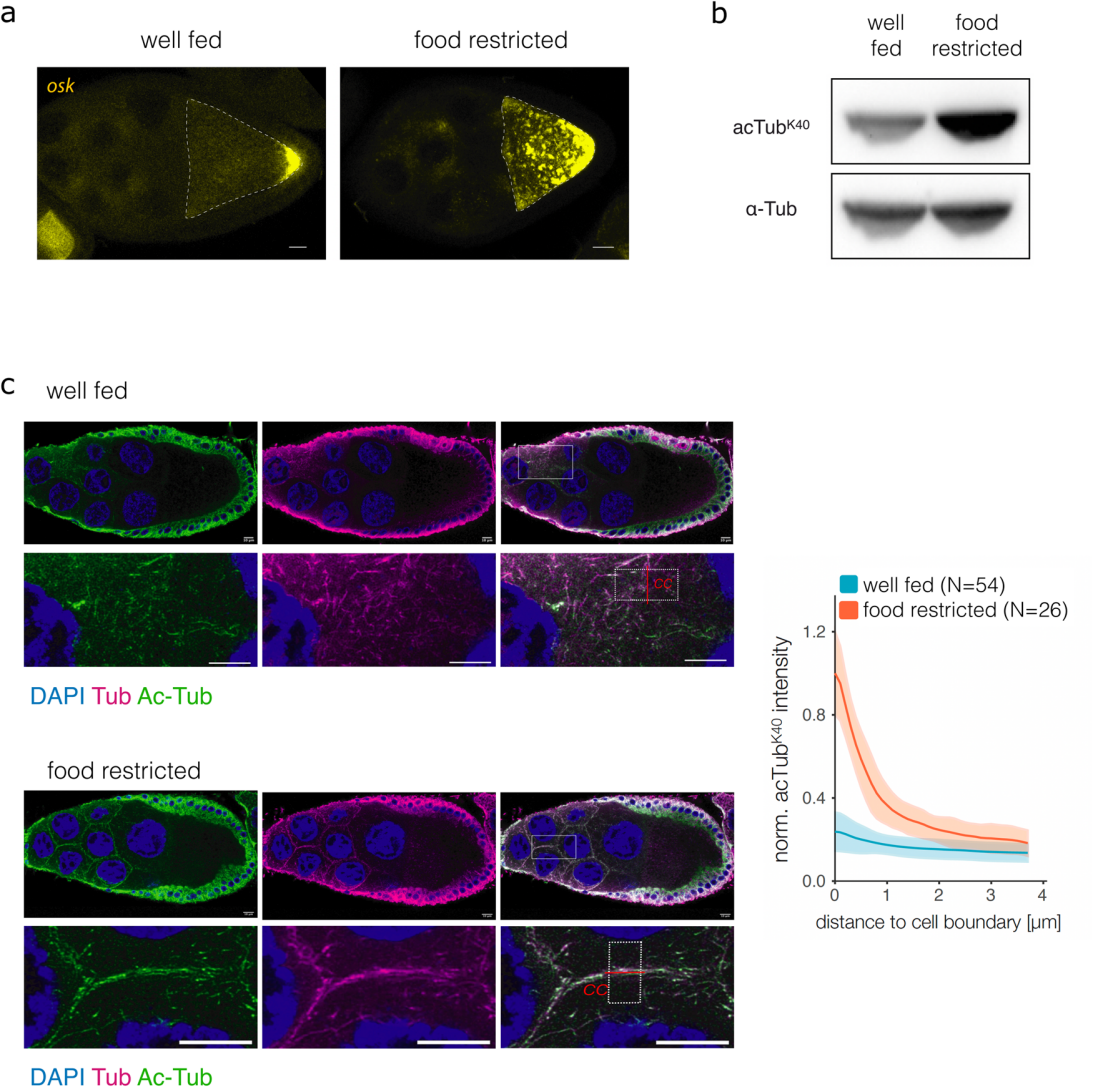
α-Tubulin acetylation affects mRNA transport in the developing oocyte. **a** *oskar* mRNA localization in egg chambers from well-fed and nutritionally deprived flies visualized by smFISH. The oocyte is highlighted by a dashed line. **b** Western blot analysis of α-tubulin K40 acetylation in ovarian lysates from well-fed and nutritionally deprived flies. Band size: ~50 kDa for tubulin and acetylated tubulin. **c** Acetylation of α-tubulin in egg chambers from well-fed and nutritionally deprived flies stained for tubulin (tub), acetylated α-tubulin K40 (Ac-Tub), and DAPI. Lower panels show a magnification of the rectangular area highlighted in the upper panels on the right (merged signals). Graph shows a quantification of the fluorescence signal at cell-to-cell boundaries (cc, red line) between nurse cells in ovaries of well-fed (*N* = 54) and  food-restricted (*N* = 26) flies. The fluorescent profile of a squared area (within white dashed line) parallel to the cell boundary was used for quantification, and fluorescence intensity was normalized to the maximum observed intensity. Shaded areas represent SD. Scale bar: 10 μm.

Upon nutrient deprivation, egg chambers form large reticulated P-bodies that contain maternal mRNAs, including *oskar* mRNA^1,2^ (Fig. 1a). Formation of P-bodies is promoted by insulin signaling-mediated alterations in microtubule dynamics and organization, and specifically the depletion of cytoplasmic microtubules in egg chambers^1,2^. We found that α-tubulin in *Drosophila* ovarian lysates is hyperacetylated at K40 upon nutritional stress, provoked by withdrawal of the standard protein-rich fly diet (Fig. 1b). While nutritional stress results in the depletion of the cytoplasmic pool of microtubules, an accumulation of microtubules has been observed in the cortical region of nurse cells^2^. Here, we found that the cortically enriched microtubules are hyperacetylated in nutrient-deprived egg chambers (Fig. 1c and Supplementary Fig. 1). Thus, hyperacetylation of α-tubulin at K40 correlates with the enrichment of cortical microtubules in nurse cells and the formation of P-bodies.

**Fig. 2:**
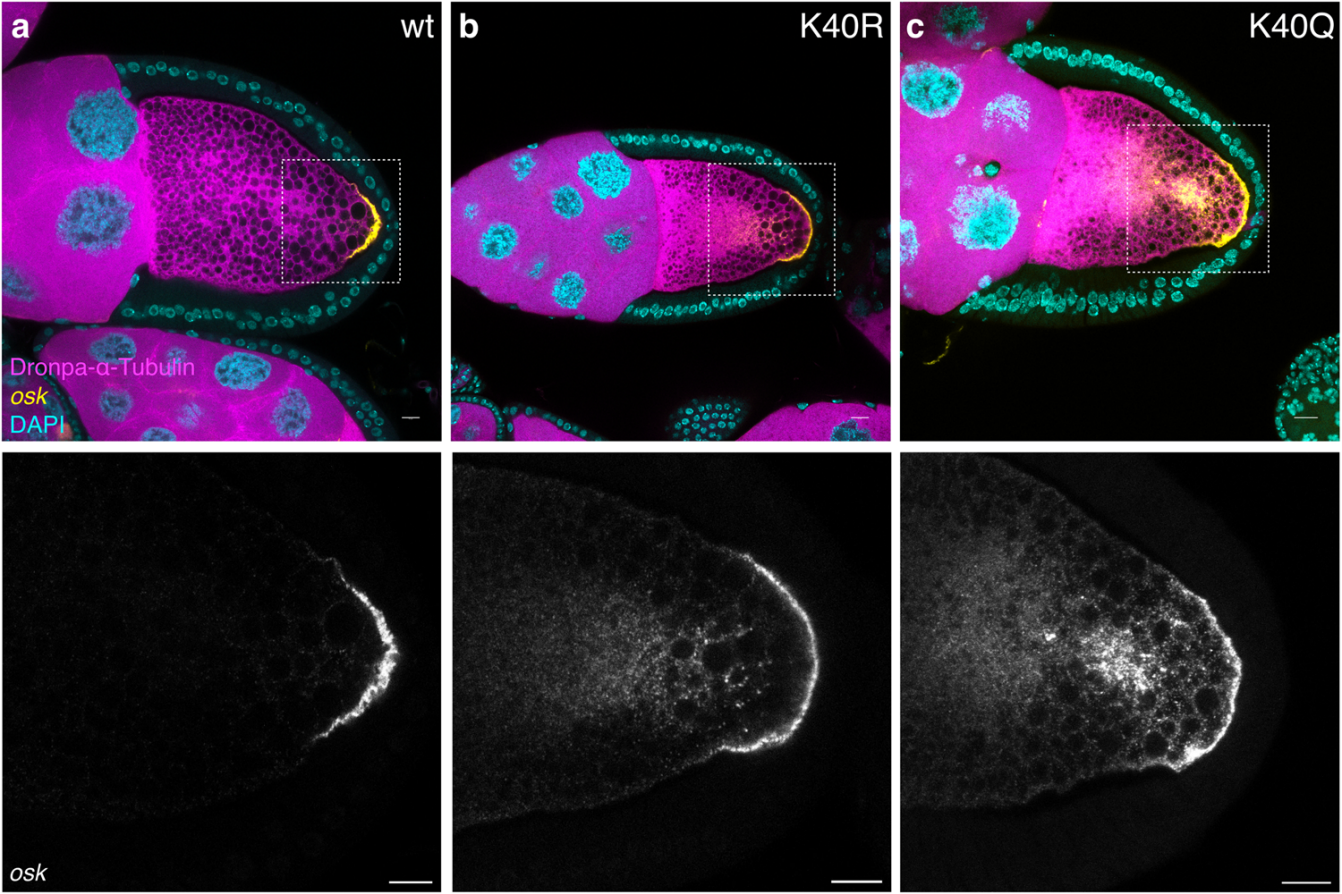
Acetylation mimetic α-tubulin K40Q affects the localization of *oskar* mRNA. **a**–**c** Representative confocal images of egg chambers expressing Dronpa-α-tubulin wild-type, K40R, and K40Q mutants. *oskar* mRNA localization was visualized by smFISH. Scale bar: 10 μm.

To determine the role of α-tubulin acetylation at K40, we made use of transgenic flies that overexpress either wild-type, acetylation deficient (K40R), or acetylation mimetic (K40Q) α-tubulin^17^. The α-tubulin was tagged with the photo-switchable fluorescent protein Dronpa^18^ to visualize tubulin in vivo. At mid-oogenesis, *oskar* mRNA localizes to the posterior of the oocyte. Overexpression of either wild-type or acetylation deficient K40R α-tubulin did not affect *oskar* mRNA localization (Fig. 2a, b), whereas the acetylation mimetic mutant α-tubulin K40Q did, resulting in substantial *oskar* mRNA accumulation throughout the ooplasm, with only a smaller proportion being localized to the posterior pole (Fig. 2c). The *oskar* mRNA levels were similar for the three lines, as quantified by qRT-PCR (Supplementary Fig. 2). The dominant effect of α-tubulin K40Q suggests that changes in α-tubulin dynamics caused by mimicking hyperacetylation are involved in the accumulation of *oskar* mRNPs into P-bodies, as seen during nutritional stress.

### HDAC1 modulates microtubule dynamics and mRNA transport

**Fig. 3:**
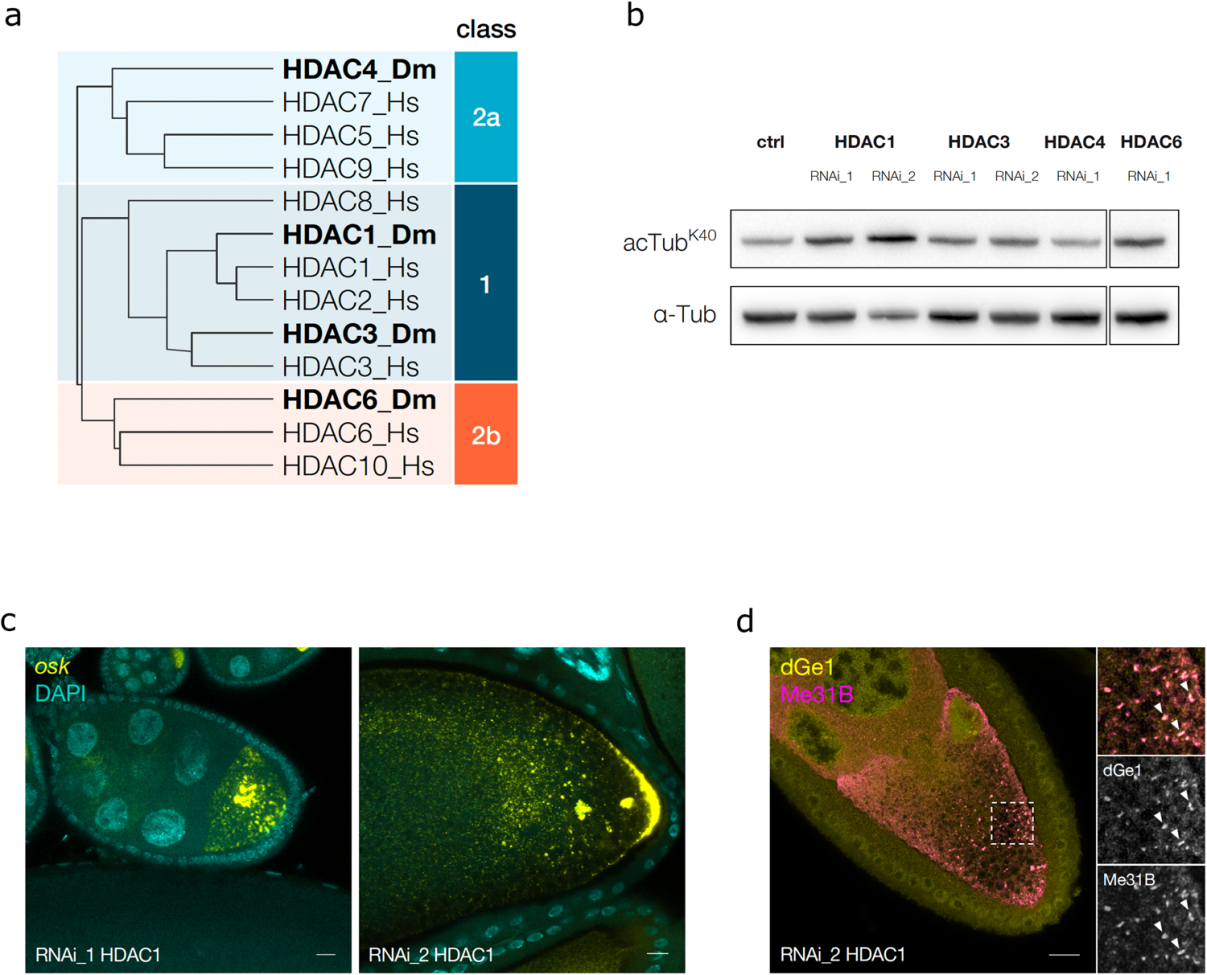
HDAC1 RNAi affects α-tubulin acetylation and mRNP transport. **a** Phylogenetic analysis of class 1, 2a, and 2b HDAC proteins from Homo sapiens (Hs) and *Drosophila melanogaster*^40^ using Clustal Omega. (UniprotIDs for Hs: Q13547, Q92769, O15379, Q9UQL6, Q9UBN7, Q8WUI4, Q9BY41, Q9UKV0, Q969S8; for Dm: Q94517, Q7KTS4, Q9VYF3, Q86NK9). **b** Western blot analysis of α-tubulin K40 acetylation from ovaries in which HDAC1, HDAC3, HDAC4 and HDAC6 were knocked down using RNAi in the germline. Band size: ~50 kDa for tubulin and acetylated tubulin (**c**) *oskar* mRNA localization visualized using smFISH in HDAC1 knockdown egg chambers using two different RNAi lines. **d** Immunofluorescence of dGe1 and Me31B accumulating in P-bodies in an HDAC1-knockdown egg chamber. Scale bar: 10 µm.

Given cytoplasmic HDACs can deacetylate α-tubulin in vitro and in vivo^11–13^. we assayed a panel of *Drosophila* class 1, 2a, and 2b HDACs for their impact on α-tubulin acetylation under well-fed conditions (Fig. 3a). We found that germline-specific knockdown of HDAC1 using two different RNAi lines substantially increased α-tubulin K40 acetylation (Fig. 3b), as seen during nutritional stress (Fig. 1b). Knockdown of other HDACs (Supplementary Fig. 3), including HDAC6, which has been shown to be the main mammalian α-tubulin deacetylase^11,12^, showed only minor effects on α-tubulin K40 acetylation levels in the *Drosophila* female germline (Fig. 3b).

We also found that *oskar* mRNA transport was strongly perturbed upon HDAC1 knockdown, as evident from the aggregation of *oskar* mRNA in large puncta, resembling P-bodies, in the oocyte cytoplasm (Fig. 3c). Indeed, when we investigated the subcellular localization of the mRNA granule marker proteins Me31B and dGe1, we found that despite the lack of nutritional stress, both proteins aggregated in P-bodies (Fig. 3d). Furthermore, knockdown of HDAC3, HDAC4, and HDAC6 did not lead to aggregation of *oskar* mRNPs under well-fed conditions, indicating the phenotype is specific to HDAC1 (Supplementary Fig. 4). This suggests that HDAC1 maintains low levels of α-tubulin acetylation in the *Drosophila* female germline under well-fed conditions, presumably promoting the microtubule dynamics required for normal mRNP transport.

### *Drosophila* HDAC1 directly deacetylates α-tubulin

**Fig. 4:**
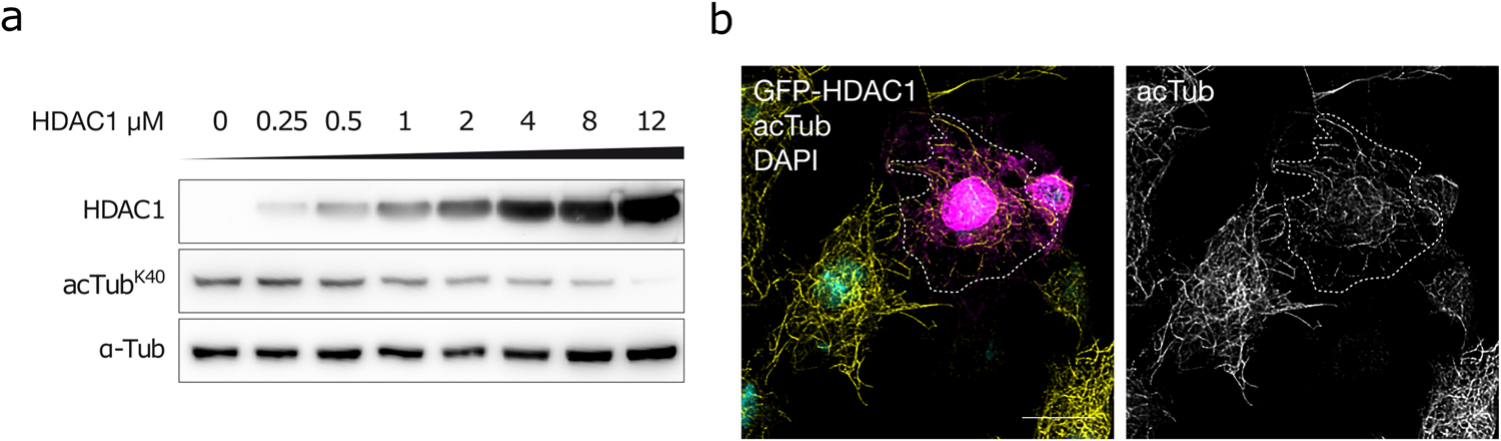
HDAC1 deacetylates α-tubulin in vitro and in vivo. **a** In vitro deacetylation assay using indicated amounts of recombinant HDAC1 with 1 μM porcine tubulin, incubated for 2 h at 25 °C and analyzed by western blot. Band size: ~50 kDa for tubulin and acetylated tubulin, and ~75 kDa for recombinant HDAC1. **b** S2R+ cells transiently transfected with GFP-HDAC1, stained 24 h post transfection for acetylated α-tubulin K40 and DAPI. A transfected cell is highlighted by the dashed line. Scale bar: 10 μm.

To test whether *Drosophila* HDAC1 directly deacetylates α-tubulin we produced recombinant HDAC1 protein using a baculovirus-mediated expression system in insect cells. When incubated with tubulin purified from porcine brain, we found that HDAC1 was able to reduce the level of acetylated α-tubulin in a concentration-dependent manner, showing that *Drosophila* HDAC1 can efficiently deacetylate α-tubulin (Fig. 4a). Overexpression of HDAC6 has been shown to reduce the amount of acetylated α-tubulin in human cells, while other HDACs showed no effect on α-tubulin acetylation levels^11,12^. We found that overexpression of GFP-tagged *Drosophila* HDAC1 in S2R+ cells resulted in substantially reduced acetylation of α-tubulin K40 in transfected compared to untransfected cells (Fig. 4b and Supplementary Fig. 5a). This indicates that, in contrast to human HDAC1^11,12^, *Drosophila* HDAC1 directly deacetylates α-tubulin, both in vitro and in vivo.

### Phosphorylation-mediated control of microtubule acetylation

**Fig. 5:**
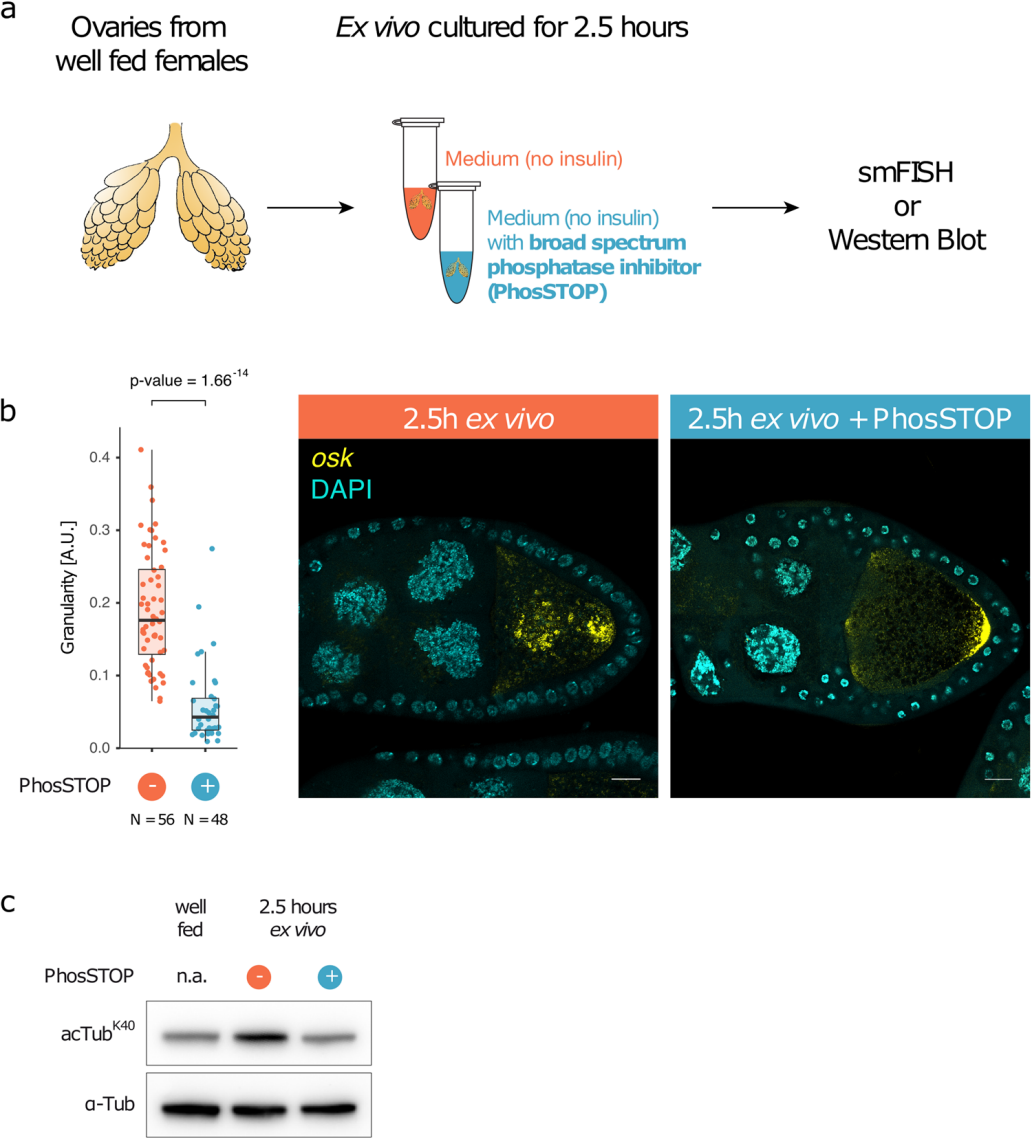
Phosphorylation events act upstream of P-body formation and α-tubulin acetylation. **a** Schematic representation of ex vivo culturing assay of ovaries. Ovaries from well-fed females were dissected manually and transferred to medium or medium supplemented with a broad-spectrum phosphatase inhibitor (PhosSTOP). After an incubation for 2.5 h, ovaries were subjected to staining using smFISH or analysis by western blot. **b** smFISH staining for *oskar* mRNA in egg chambers cultured with or without PhosSTOP for 2.5 h ex vivo. Statistical significance was calculated using Welch two-sample *t* test. **c** Acetylation of α-tubulin K40 from ovaries cultured with or without PhosSTOP for 2.5 h ex vivo was analyzed by western blot (band size: ~50 kDa for tubulin and acetylated tubulin). Scale bar: 10 µm.

Insulin/TOR signaling has been shown to transmit information about nutritional status to *Drosophila* follicle cells and to regulate the starvation response^1^. However, it is unclear which signaling pathways mediate the nutritional stress response within the germline. Supplementing ex vivo cultured egg chambers with insulin suppresses the formation of P-bodies^2^. We therefore wondered if P-body formation is sensitive to changes in the phosphorylation status of proteins, given phosphorylation underlies the signaling cascade of the insulin/TOR pathway. When cultured for 2.5 h ex vivo without insulin (Fig. 5a), egg chambers showed a typical starvation response, with *oskar* mRNA accumulating in P-bodies (Fig. 5b), as is seen in vivo (Fig. 1a). However, the addition of a broad-spectrum phosphatase inhibitor (PhosSTOP) substantially reduced the formation of *oskar* mRNA containing P-bodies (Fig. 5b), suggesting that phosphorylation is required for the nutritional stress response. Moreover, hyperacetylation of α-tubulin caused by ex vivo culturing of egg chambers without insulin could also be suppressed by the addition of the phosphatase inhibitor (Fig. 5c). Taken together, this suggests that phosphorylation events acting upstream of α-tubulin acetylation/deacetylation regulate microtubule dynamics and subsequent formation of P-bodies in response to nutrition.

### Phosphorylation dynamics upon nutritional stress

**Fig. 6:**
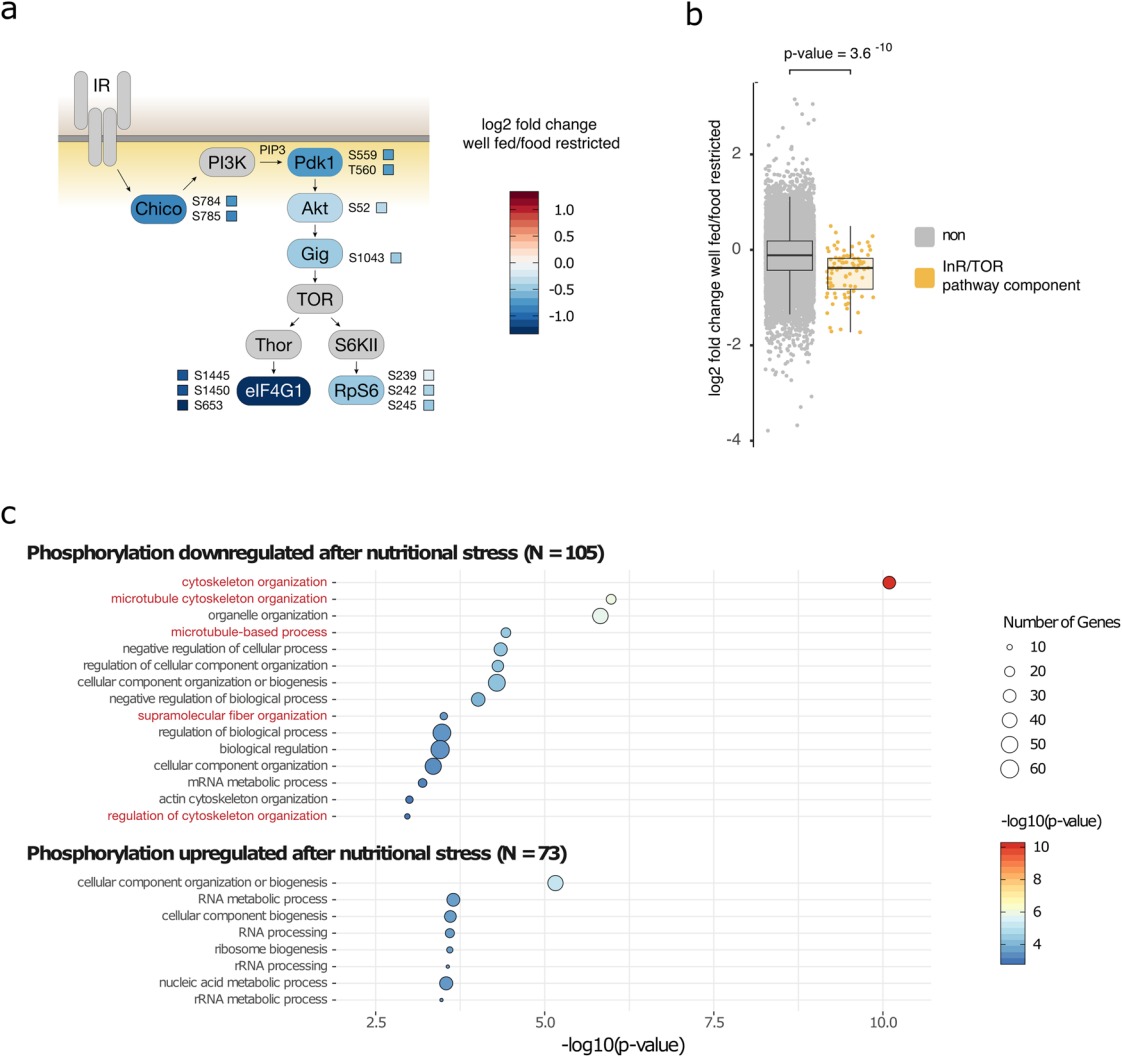
Nutritional stress-induced changes in the phosphoproteome of *Drosophila* egg chambers. **a** Schematic representation of the insulin/TOR signaling pathway. Individual phosphorylation sites of relevant proteins which were analyzed by quantitative phosphoproteomics are highlighted. **b** Quantification of all phosphorylation sites of proteins in the insulin/TOR signaling pathway. Statistical significance was calculated using Welch two-sample *t* test. **c** GO-term enrichment analysis of proteins whose phosphorylation sites were significantly down- or upregulated in response to nutritional stress (Benjamini–Hochberg corrected).

In order to identify the signaling events involved in the nutritional stress response in vivo, we analyzed the phosphorylation state of proteins from well-fed and nutrient-deprived egg chambers. TiO_2_ enrichment of phosphopeptides followed by quantitative mass spectrometry was used to quantitatively monitor 16,975 phosphorylation sites in 2822 proteins (Supplementary Data 2). We found that the phosphorylation sites of insulin/TOR signaling-related proteins were significantly less phosphorylated upon nutritional stress (Fig. 6a, b and Supplementary Data 2) consistent with somatic insulin and TOR signaling transmitting information about the nutritional status to the germline and to regulate the starvation response^1^.

We asked which biological and molecular functions are associated with proteins whose phosphorylation states were altered upon nutritional stress. Proteins whose phosphorylation sites were significantly downregulated (*P* value < 0.05) showed an enrichment of gene ontology (GO) terms associated with cytoskeleton organization, microtubule cytoskeleton organization, microtubule-based processes, supramolecular fiber organization, and regulation of cytoskeleton organization (Fig. 6c and Supplementary Data 3). Proteins whose phosphorylation sites were significantly upregulated showed no significant enrichment of GO terms related to the cytoskeleton. Thus, post-translational phosphorylation modifications play a role in transmitting nutritional status information to regulate cytoskeletal dynamics in the *Drosophila* female germline.

### HDAC1 activity during nutritional stress is regulated by phosphorylation

**Fig. 7:**
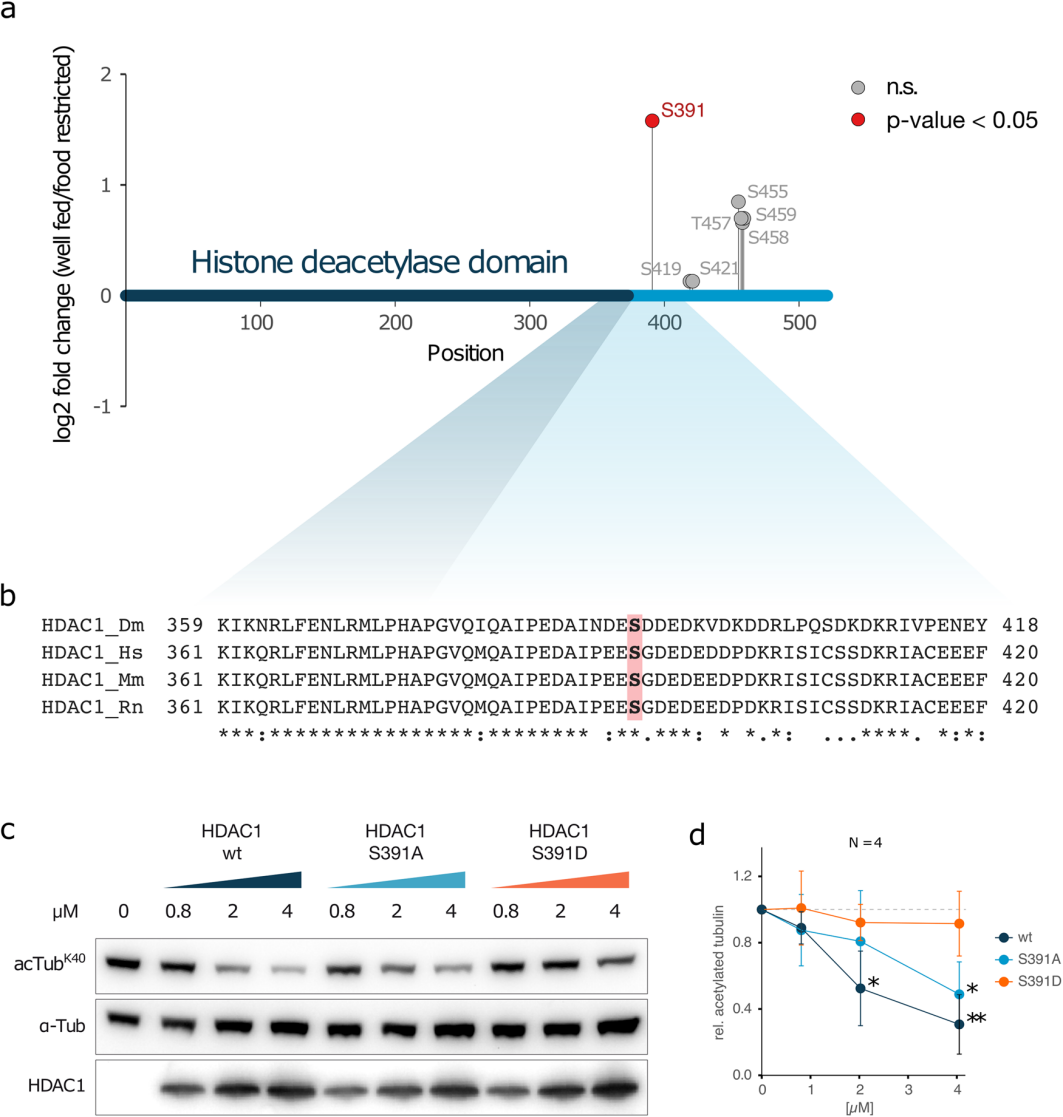
HDAC1 phosphorylation and the effect on α-tubulin deacetylation activity. **a** Visualization of *Drosophila* HDAC1 protein and the phosphorylation sites identified by quantitative phosphoproteomics. **b** Alignment of the conserved region around S391 of HDAC1 from *Drosophila melanogaster*^40^, Homo sapiens (Hs), Mus musculus (Mm), and Rattus norvegicus (Rn). **c** Western blot analysis of in vitro deacetylation assay using recombinant wild type, mutant S391A and S391D HDAC1 at indicated concentrations and purified tubulin (1 μM), incubated for 1 h at 25 °C. Band size: ~50 kDa for tubulin and acetylated tubulin, and ~75 kDa for recombinant HDAC1. **d** Quantification of western blot analysis of in vitro deacetylation assays as in **c**) (*N* = 4). Error bars represent SD. Statistical significance was calculated using Welch two-sample *t* test: *P value < 0.05, **P value < 0.01.

HDACs are themselves regulated by post-translational modifications, such as phosphorylation, to either modulate enzymatic activity, dimerization or subcellular localization^14–16^. We identified seven phosphorylation sites in HDAC1 (Fig. 7a), of which only Serine 391 (S391) showed a significant increase in phosphorylation in response to nutritional stress compared to the remaining 6 phosphorylation sites, which were not significantly altered. S391 is located in a disordered region C-terminal to the histone deacetylase catalytic domain and the residue is highly conserved from fly to mouse, rat and human (Fig. 7b). In humans, the corresponding sites in HDAC1 (S393) and HDAC2 (S394) have been shown to be phosphorylated^14^. Phosphorylation of S421/S423 in mammalian HDAC1 enzymes regulates catalytic activity^19,20^. The effect of phosphorylation at S393 and at sites other than S421/S423 in mammalian HDAC1 is presently unclear.

To study the effect of S391 phosphorylation in *Drosophila* HDAC1, we produced phosphorylation-deficient (S391A) and phosphorylation mimetic (S319D) HDAC1 proteins using a baculoviral expression system. We found that phosphorylation-deficient HDAC1 S391A had a deacetylation activity comparable to wild-type HDAC1 on purified tubulin (Fig. 7c, d). The phospho-mimetic HDAC1 S391D failed to deacetylate α-tubulin K40, suggesting that, in *Drosophila*, phosphorylation of S391 suppresses the enzymatic activity of HDAC1 towards α-tubulin.

Consistent with the in vitro data, wild-type and GFP-HDAC1-S391A overexpression in S2R+ cells significantly reduced the level of acetylated α-tubulin K40 in transfected cells compared to non-transfected neighboring cells (Supplementary Fig. 5a). In contrast, phospho-mimetic GFP-HDAC1-S391D was unable to reduce the level of acetylated α-tubulin, consistent with the diminished activity of phospho-mimetic HDAC1 S391D in vivo. Phosphorylation has also been shown to regulate nucleocytoplasmic trafficking of HDACs, which might influence acetylation dynamics due to reduced substrate availability^15^. However, neither the phospho-mimetic (S391D) nor the phospho-deletion (S391A) mutation altered the nuclear to cytoplasmic ratio of GFP-HDAC1 in cultured cells (Supplementary Fig. 5b). Taken together, our data show that HDAC1 is dephosphorylated at S391 in response to nutritional stress, which decreases its ability to deacetylate α-tubulin.

Surprisingly, we found no substantial difference between wild-type HDAC1, HDAC1 S391A, and HDAC1 S391D in an assay that used short artificial peptide substrates to measure deacetylase activity (Supplementary Fig. 6a). This suggests that rather than directly influencing the enzymatic activity of the deacetylase domain, phosphorylation at S391 regulates substrate recognition or release. The disordered C-terminus of HDAC1 and its phosphorylation state may regulate substrate recognition, an idea consistent with a recent report suggesting a role for the unstructured N-terminus of mammalian HDAC6 in α-tubulin recognition^21^. We found substantial differences in the amount of copurified α-tubulin when preparing the different phospho-mutant HDAC1s from insect cells (Supplementary Fig. 6b). Compared to wild-type HDAC1, HDAC1 S391A copurified less and HDAC1 S391D much more α-tubulin, suggesting that the phosphorylation status of S391 plays a role in regulating the binding and/or release of the α-tubulin substrate.

## Discussion

Microtubule dynamics are critical for mRNA transport and mRNP granule formation in the *Drosophila* female germline^1,22^. During nutritional stress, α-tubulin and microtubules are depleted from the cytoplasm and accumulate cortically. The loss of cytoplasmic microtubules may result in the aggregation of mRNPs and the formation of P-bodies.

Here, we show that hyperacetylation of α-tubulin K40 correlates with altered microtubule dynamics and the formation of P-bodies during nutritional stress in the *Drosophila* female germline (Fig. 1a, b). A similar mechanism operates in mammalian cells, where nutritional stress has been shown to induce the hyperacetylation of α-tubulin, a phenomenon crucial for the formation of autophagosomes and starvation-induced autophagy^9^. Since the starvation response in egg chambers also leads to the induction of autophagy^23^, it is possible that microtubule hyperacetylation promotes recruitment of autophagy-inducing factors during the starvation response. Additionally, it is possible that in mammalian cells, as in *Drosophila* egg chambers, changes in microtubule dynamics contribute to mRNP aggregation and stress granule formation upon nutrient deprivation.

**Fig. 8:**
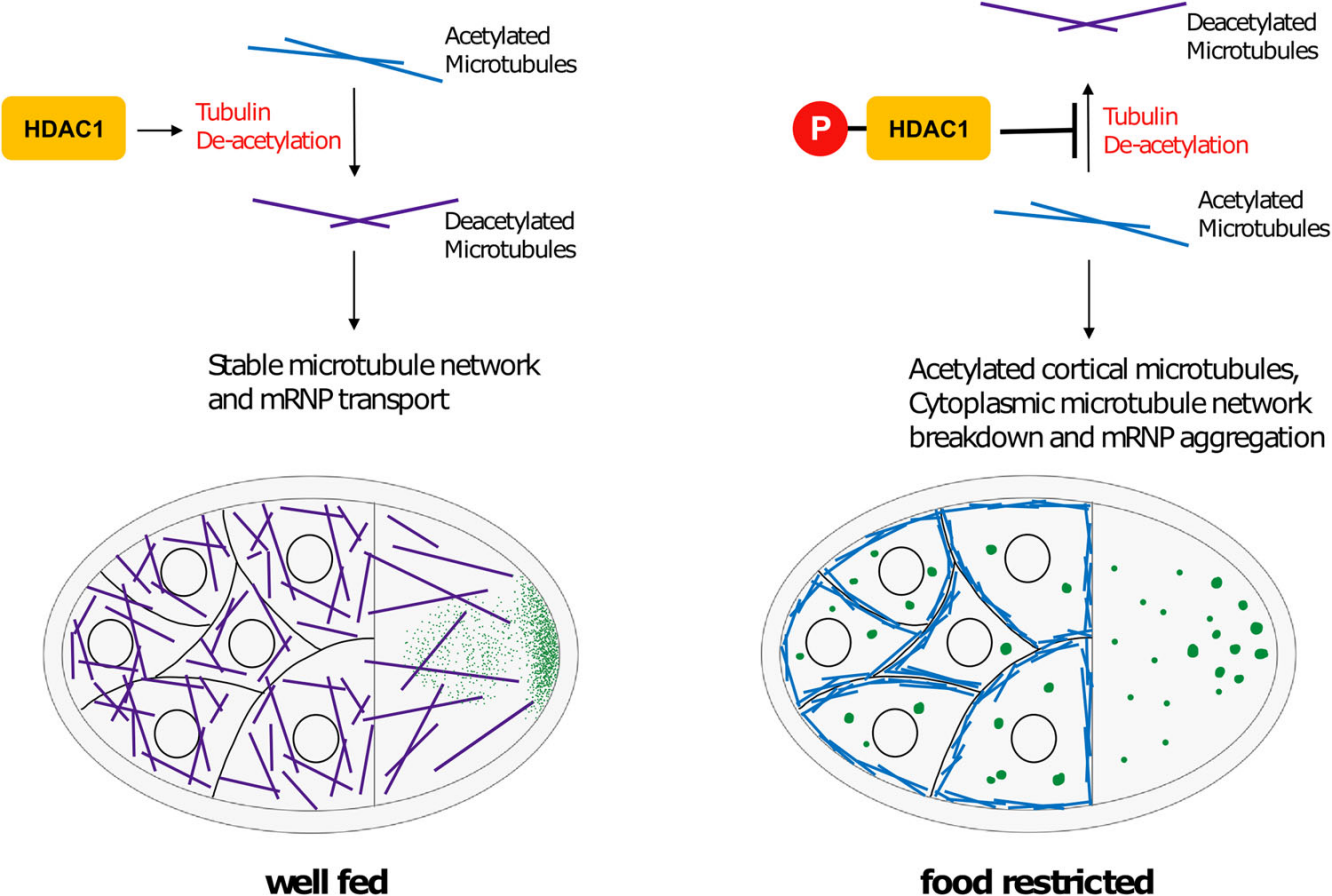
Model for regulation of starvation response by HDAC1. In well-fed flies, HDAC1 maintains low levels of acetylated microtubules in the germline. Upon food deprivation, phosphorylation of HDAC1 reduces its ability to deacetylate microtubules, resulting in an accumulation of acetylated microtubules near the cell cortex.

Further, we show that acetylation mimetic α-tubulin K40Q can phenocopy the effects of nutritional stress (Fig. 2c). Acetylation of α-tubulin at K40 is known to alter microtubule dynamics by enhancing the flexibility of microtubules, thereby protecting them from mechanical stresses and making them more long-lived^7^. Thus, we propose a model in which nutritional stress-induced hyperacetylation of α-tubulin alters microtubule organization, depleting them from the cytoplasmic pool of dynamic microtubules, which then leads to the aggregation of mRNPs (Fig. 8).

Class II HDACs, such as the cytoplasmic HDAC6, have been shown to deacetylate α-tubulin^11–13^. Here, we identified class I HDAC1 as the deacetylase principally responsible for the deacetylation of α-tubulin in the *Drosophila* female germline (Fig. 3b). We further show that *Drosophila* HDAC1, in contrast to mammalian HDAC1^11^, directly deacetylates α-tubulin in vitro and in vivo (Fig. 4a, b). Why HDAC1 is more involved in the regulation of α-tubulin K40 acetylation than HDAC6 in the *Drosophila* female germline remains to be clarified. *Drosophila* HDAC1 and HDAC6 have anticorrelated temporal expression patterns during developmental stages: HDAC1 is more highly expressed in the early developmental stages, whereas HDAC6 expression is highest in the adult fly^24^. Therefore, specific HDACs might carry out similar functions and have overlapping substrates throughout developmental stages.

Phosphorylation-mediated regulation of HDACs has been shown to affect their enzymatic activity, subcellular localization and dimerization^14–16^. Quantitative phosphoproteomics revealed that HDAC1 S391 is significantly phosphorylated in response to nutritional stress (Fig. 7a), and the phospho-mimetic mutant HDAC1 S391D shows substantially reduced α-tubulin K40 deacetylation activity in vitro and in vivo compared to wild type or phospho-deletion S391A HDAC1 (Fig. 7c, d and Supplementary Fig. S5a). However, the phospho-mimetic mutant HDAC1 S391D could deacetylate a short peptide substrate as well as the wild-type enzyme or the phosphorylation-deficient mutant HDAC1 S391A (Supplementary Fig. 6a). This suggests a regulatory mechanism which involves the phosphorylation of S391 for substrate recognition and/or release. Indeed, removal of the N-terminal microtubule-binding domain of HDAC6 has been shown to have no effect on the deacetylase activity towards short peptides, but was crucial for the recognition of α-tubulin^21^. We found substantial differences in the amount of copurified α-tubulin when preparing the different phospho-mutant HDAC1s from insect cells (Supplementary Fig. 6b). Compared to wild-type HDAC1, HDAC1 S391A copurified less and HDAC1 S391D much more α-tubulin, suggesting that the phosphorylation status of S391 within the disordered C-terminus plays a role in regulating the binding and/or release of the α-tubulin substrate.

Protein kinase CK2, and PKA and Aurora kinases have been shown to phosphorylate human class I HDACs^19,25^. When analyzing our phosphoproteomics data with respect to known kinase consensus motifs, we found a significant increase in phosphorylation of CK2 consensus motifs upon nutritional stress (Supplementary Fig. 7a). Furthermore, S391 of *Drosophila* HDAC1 is embedded in a predicted target of the CK2 consensus sequence (X-(S/T)-X-X-(D/E), whose corresponding site in human HDAC1 has been shown to be phosphorylated by CK2 in vitro^26^. CK2 has been shown to play important roles in carbohydrate metabolism, where it regulates insulin production and secretion, as well as the activity of enzymes involved in carbohydrate storage and metabolism in hormone-sensitive cells^27^. Hence, it was of interest to test if, in *Drosophila* egg chambers, the starvation response is also dependent on CK2-mediated phosphorylation. However, culturing ovaries ex vivo in the presence of the selective CK2 inhibitor tetrabromocinnamic acid (TBCA) did not affect the nutritional stress-induced hyperacetylation of α-tubulin (Supplementary Fig. 7b). At very high concentrations, CK2 inhibition even increased the hyperacetylation of α-tubulin K40. Thus, we found no evidence that CK2 is involved in the nutritional stress-induced phosphorylation of HDAC1 and the subsequent hyperacetylation of α-tubulin in the *Drosophila* female germline.

Taken together, our results reveal that *Drosophila* HDAC1 plays a role in nutritional status sensing, which regulates germline development. Changes in microtubule dynamics are likely controlled by a number of different signaling pathways. We found several examples of microtubule-associated proteins whose phosphorylation status was significantly altered in response to nutritional stress, such as Tau, Futsch and its interaction partner Ank2, Spectrin, mei-38 (Supplementary Fig. 8). We also found regulators of microtubule dynamics, such as Ensconsin, Stathmin, BicC and Tral, and other cytoskeleton-associated proteins, such as Hts, to be less phosphorylated. For several of these (e.g., Tau, Ensconsin, and Stathmin), phosphorylation has been shown to have a function in the regulation of binding to microtubules^28–30^.

## Methods

### Fly stocks

*w*^*1118*^ was used as wild type. Oregon-R (Bloomington Stock 5#) was used for mass extraction of ovaries for phosphoproteomics. RNAi against HDAC1 (RNAi_1: Bloomington Stock #36800; RNAi_2: Bloomington Stock #33725), HDAC3 (RNAi_1: Bloomington Stock #64476; RNAi_2: Bloomington Stock #34778), HDAC4 (RNAi_1: Bloomington Stock #34774; RNAi_2: Bloomington Stock #28549), HDAC6 (RNAi_1: Bloomington Stock #34072) and control RNAi (Bloomington Stock #35573) were driven by MTD-Gal4 (Bloomington Stock # 31777). K40R and K40Q α-tubulin expressing lines were from ref. ^17^. YFP-DCP1 fly line was from ref. ^31^.

### Antibodies and reagents

The following antibodies were used: mouse anti-α-tubulin (Sigma, T5168), mouse anti-acetylated-α-tubulin (Sigma, T7451), mouse anti-Me31B (from ref. ^32^), rat anti-dGe1 (from ref. ^33^), rabbit anti-GFP antibody-Alexa Fluor 488 conjugate (Invitrogen, A-21311) and rabbit anti-HDAC1 (gift of Jürg Müller).

### Fluorescent in situ hybridization

Ovaries from 2-day-old females, well-fed or protein-deprived, were dissected into 2% v/v PFA (Electron Microscopy Sciences, #15710), 0.05% v/v Triton X-100 (Sigma, P1379) in PBS (pH 7.4) and were fixed for 20 min. After two washes with PBSTX (0.1% Triton x-100 in PBS), ovaries were pre-hybridized 200 µl hybridization buffer (300 mM NaCl, 30 mM Sodium citrate pH 7.0, 15 v/v% ethylene carbonate, 1 mM EDTA, 50 µg/ml heparin, 100 µg/ml salmon sperm DNA, 1 v/v% Triton X-100) for 10 min at 42 °C. All probes were labeled as described previously^34^. In total, 50 µl of pre-warmed probe mixture (12.5–25 nM/individual oligonucleotide) was added and hybridization was allowed to proceed for 2 h at 42 °C. Samples were washed by pre-warmed hybridization buffer, hybridization buffer: PBSTX 1:1 mixture, PBSTX, each for 10 min at 42 °C. Finally, pre-warmed PBSTX was added and the sample was allowed to cool down to room temperature. Ovaries were mounted in 80 v/v% 2,2-thiodiethanol in PBS. Stacks of images were acquired on a Leica TCS SP8 confocal microscope using a 63 × 1.4 NA oil immersion objective. Images were processed using ImageJ (http://rsb.info.nih.gov/ij/). Granularity (area covered by *oskar* mRNA positive P-bodies/total oocyte area) was used as a quantitative measure for P-body formation.

### Immunostaining of egg chambers

For the visualization of acetylated α-tubulin, ovaries from 2-day-old well-fed females were dissected into ice-cold methanol and fixed for 20 min at −20 °C. Ovaries were subsequently washed and rehydrated with PBS for 1 h, blocked in 10% goat serum in PBS for 1 h, and stained overnight at 4 °C with mouse anti-acetylated-α-tubulin antibody (1:500). After washing, ovaries were incubated with a secondary antibody donkey anti-mouse-Cy3 (1:500) (Jackson ImmunoResearch 715-165-151) and stained with DAPI. For visualization of Me31B, dGe1, and DCP1, ovaries were dissected into 2% v/v PFA (Electron Microscopy Sciences, #15710), 0.05% v/v Triton X-100 (Sigma, P1379) in PBS (pH 7.4) and fixed for 20 min. After washes in PBST (0.1% Tween20 in PBS), ovaries were permeabilized in 1% Triton X-100 in PBS for 1 h, blocked with 10% goat serum in PBS and stained with primary antibodies (1:500) in blocking buffer. After washing, ovaries were incubated with a secondary antibody, donkey anti-mouse-Cy3 (1:500) (Jackson ImmunoResearch, 715-165-151) for Me31B and dGe1, and anti-GFP antibody-Alexa Fluor 488 conjugate (1:500) (Invitrogen, A-21311) for YFP-DCP1. The samples were then stained with DAPI.

Stacks of images were acquired on a Leica TCS SP8 LIGHTNING confocal microscope using a 63 × 1.4 NA oil immersion objective. Images were processed and analyzed using ImageJ (http://rsb.info.nih.gov/ij/).

### Immunostaining of S2R+ cells

HDAC1 was amplified from *w*^*1118*^ cDNA using the primer pair CACCATGCAGTCTCACAGCAAAAAGC/ TCAAATGTTGTTCTCCTTGGCG and inserted into pENTR/D-TOPO (Invitrogen). S391A and S391D mutations were introduced by site directed mutagenesis. The inserts were then cloned into the destination vector pAGW (The Drosophila Gateway Vector Collection) using the Gateway LR Clonase II (Thermo, 11791100). pA-GFP-HDAC1 wt/S391A/S391D were transfected into S2R^+^ cells using Effectene (Qiagen, 301425). 24 h post transfection, cells were fixed with ice-cold methanol for 15 min at −20 °C. After permeabilized with 0.1% Triton X-100, cells were blocked with 10% goat serum in PBS and stained with mouse acetylated-α-tubulin. After washing, cells were incubated with a secondary antibody donkey anti-mouse-Cy3 (Jackson ImmunoResearch 715-165-151), and stained with DAPI. Stacks of images were acquired on a Leica TCS SP8 LIGHTNING confocal microscope using a ×63 1.4 NA oil immersion objective. Images were processed and analyzed using ImageJ (http://rsb.info.nih.gov/ij/).

### Ex vivo culturing of egg chambers

Ovaries from *w*^*1118*^ females kept on yeast paste were extracted and kept in Schneider’s *Drosophila* Medium (Gibco, 21720-024) supplemented with 10% FBS for 2.5 h at room temperature. For the inhibition of phosphatases, the medium was supplemented with PhosSTOP (Roche, 4906845001), for the inhibition of CK2 the medium was supplemented with indicated amounts of TBCA (Sigma, SML0854). DMSO was used as a control. Ovaries were harvested and subjected to analysis by western blot or fluorescent in situ hybridization.

### Deacetylation of tubulin and short peptides

*Drosophila* HDAC1 (wt/S391A/S391D) was cloned into pCoofy41 using SLIC cloning^35^. HDAC-TwinStrep was expressed in Sf21 cells using a baculovirus-mediated expression. Cell pellets were resuspended in Lysis Buffer (50 mM Tris, 150 mM NaCl, 1 mM EDTA, 0.01% NP40, cOmplete Mini EDTA-free Protease inhibitor cocktail (Roche) and Benzonase) and lysed in a Microfluidizer (M-110L, Microfluidics). The lysate was cleared by centrifugation at 40,000×*g* for 30 min at 4 °C. The supernatant was loaded onto a StrepTrap HP column (GE Healthcare), washed with Binding Buffer (50 mM Tris, 150 mM NaCl, 1 mM EDTA), and eluted with 2.5 µM desthiobiotin in Binding Buffer. The eluted protein was supplemented with 5% glycerol. Single-use aliquots were flash frozen in liquid nitrogen and stored at −80 °C.

For assaying the α-tubulin deacetylase activity of recombinant HDAC1, 1 µg tubulin purified from porcine brain (Cytoskeleton, T240) was mixed with 0.5 µg/µl BSA in assay buffer (50 mM Tris, 150 mM NaCl, 1 mM EDTA, 3 mM KCL, 1 mM MgCl, 10% glycerol) and supplemented with indicated amounts of HDAC1-TwinStrep in a 20 µl reaction. After incubation for 1 h at 25 °C, acetylation of α-tubulin was analyzed by western blot, probing against acetylated α-tubulin. Acetylated α-tubulin levels were normalized to total α-tubulin.

For the peptide deacetylation assay, recombinant HDAC-TwinStrep wild type, S391A or S391D activity was measured using a fluorimetric activity assay (Fluor de Lys, Enzo Life Sciences, BML-AK500-0001) according to the manufacturer instructions.

### Quantitative phosphoproteomics of well-fed and nutrition-deprived egg chambers

Freshly eclosed Oregon-R flies were kept in cages for 1–3 days at 25 °C on protein-rich diet (yeast paste on apple juice-agar plates). To provoke the nutritional stress, yeast paste was removed after 3 days for 4.5 h prior to the preparation of egg chambers. Flies were narcotized with CO_2_, passed through twice a grain mill (KitchenAid) with PBS and sieved through meshes with aperture sizes of 630 µm, 400 µm, 2 × 200 µm, and egg chambers were collected in a mesh with aperture sizes of 80 µm. Egg chambers were collected in 50 ml tube and washed two times with PBS. Each sample was lyzed in 200 mM HEPES buffer (pH 8) containing PhosSTOP phosphatase inhibitors (Roche), phosphatase inhibitor cocktail2 (Sigma), cOmplete Mini EDTA-free Protease inhibitor cocktail (Roche), and RapiGest SF surfactant (Waters) using a dounce homogenizer. The samples were incubated for 5 min at 70 °C in a water bath and sonicated for 20 min in a water bath. After centrifugation for 5 min at 4000 × *g*, the supernatant was transferred to a new vial and the protein concentration was determined via a bicinchoninic acid (BCA) assay. The proteins were reduced with dithiothreitol (DTT) with subsequent carbamidomethylation of cysteine residues with iodoacetamide (IAA). The modified proteins were digested with lysyl endopeptidase (Lys-C) (Wako) for 3 h at 37 °C and subsequently digested with trypsin (Promega) overnight at 37 °C. After the digestion, RapiGest SF surfactant was cleaved after the addition of 10% trifluoroacetic acid (Biomol) during 45 min at 37 °C. After centrifugation for 20 min at 4 °C at 16,000 × *g*, the supernatant was desalted on a SepPak 1 cc 50 mg cartridge (Waters). The eluate was concentrated in a vacuum centrifuge. After the addition of HEPES buffer, the pH was adjusted to 7.7 with NaOH and/or HCl.

For TMT labeling, the TMT-reagent (Thermo) was directly added to the sample and incubated for 1 h at 23 °C shaking at 300 rpm in a heating block. The reaction was stopped by the addition of 8 µl of 5% hydroxylamine and incubation for 15 min at 23 °C shaking at 300 rpm in a heating block. The sample was desalted again on a SepPak 1 cc 50 mg cartridge (Waters). The procedure was repeated in order to reach complete labeling. Prior to mixing all six samples, a test mixture was created and analyzed by LC-MS. Based on the results of this analysis, all TMT-labeled samples were mixed in a 1:1 ratio. For the mixed TMT-labeled samples, a microcolumn was packed. A plug of an Empore C8 disk (3 M) was pushed into an GELoader tip (Eppendorf). The tip was further filled with Titansphere 5 µm TiO_2_ beads (GL Science). The microcolumn was washed twice with 6% trifluoroacetic acid (TFA) in 80% acetonitrile (MeCN). The flow-through from the sample loading was collected for further analysis. Subsequently, the microcolumn was washed first with 6% TFA in 80% MeCN, second with 200 mM NaCl in 50% MeCN acidified with 0.5% TFA and third with 0.1% TFA in 80% MeCN. The sample was eluted with 5% ammonia and collected in 10% formic acid. A second elution with 0.1% TFA in 80% MeCN was collected in the same vial. The flow-through and the enriched phosphopeptides were adjusted to a basic pH with 25% ammonium just before injection. The separation was performed on an Agilent 1260 infinity high-performance liquid chromatography (HPLC) system equipped with a Waters XBridge C18; 3.5 μm; 1 × 100 mm reversed-phase column at a flow rate of 75 µl/min. The running buffers were 20 mM ammonium formate at pH 10 and 100% acetonitrile. In total, 90 one minute fractions were collected in a 96-well plate containing 25 µl 1% formic acid per well to neutralize the basic pH of the running buffer. The fractions were concentrated in a vacuum centrifuge and desalted and pooled in one step as reported previously^36^. The resulting 18 fractions from the phosphopeptide-enriched sample and the 18 fractions from the flow-through sample were separated by an UltiMate 3000 RSLC nano-LC system (Dionex). The peptides were trapped on a µ-Precolumn C18 PepMap 100 (300 µm × 5 mm, 5 µm, 100 Å) and separated on an Acclaim PepMap 100 C18 (75 µm × 50 cm, 3 µm, 100 Å) column. The analytical column was connected to a Pico-Tip Emitter (New Objective; 360 µm OD × 20 µm ID; 10-µm tip). The LC system was coupled to a QExactive plus (Thermo) via a proxeon nanoflow source. The solvents used for peptide separation were 0.1% formic acid in water and 0.1% formic acid in acetonitrile. The flow rate was 30 µl/min for trapping and 300 nl/min for the separation. The initial conditions were 2% of organic phase isocratic for 2.9 min followed by an increase to 4% in 1.1 min and 8% in 2 min. The subsequent linear gradient from 8% to 28% organic solvent was 96 min long and was followed by a 10-min gradient from 28% to 40% organic solvent. The washing of the column was at 80% organic solvent for 2 min followed by re-equilibration at 2%. Full scan spectra were acquired in positive ion mode in a mass range of 375–1200 *m/z* in profile mode with the resolution set to 70,000, with an AGC target of 3 × 10^6^ ions and a maximum ion trap fill time of 30 ms. Fragmentation spectra were recorded at a resolution of 35,000 in the orbitrap with an AGC target of 2 × 105 ions and a maximum ion trap fill time of 120 ms. A top 10 method was chosen with a precursor isolation window of 0.7 *m/z* and a fixed first mass of 100 *m/z*. The normalized collision energy was set to 32, and spectra were recorded in profile mode. Unassigned peaks as well as charge states 1, 5–8 and >8 were not chosen for fragmentation. The peptide match algorithm was set to ‘preferred’ and the dynamic exclusion to 30 s.

### Data processing for quantitative phosphoproteomics

The raw data files were processed with Thermo Proteome Discoverer version 1.4.1.14. The mass range from 126–131.3 *m/z* was excluded from the fragmentation spectra. In addition, the spectra were de-isotoped, deconvoluted, and a top N filter was applied with N set to 10 in a 100 Da window prior to the database search with Mascot version 2.5. The spectra were searched against the Uniprot database *Drosophila melanogaster* including common contaminants (22157 sequences). Semitrypsin was chosen as an enzyme with one missed cleavage allowed. Further settings were a precursor mass tolerance of 10 ppm, a fragment mass tolerance of 0.02 Da, carbamidomethylation set as fixed modification, oxidation on methionine, and phosphorylation on serine, threonine, and tyrosine set as variable modification. The quantification method included TMT as fixed modification. Percolator version 2.04 was used for false discovery rate (FDR) calculation and PhosphoRS 3.0 for calculating site probabilities for all potential phosphorylation sites. The results were exported, normalized with the vsn method^37^ and further processed by a custom analysis pipeline based on the Limma package in R/Bioconductor^38^. Protein fold changes were calculated from flow-through samples and used for the normalization of phospho-site fold changes. GO-term analysis was performed using the FlyMine database^39^.

### Statistics and reproducibility

Statistical significance of *oskar* mRNA granularity in egg chambers cultured ex vivo with or without PhosSTOP (Fig. 5) was calculated between the control group (*N* = 56 egg chambers) and PhosSTOP treated group (*N* = 48) using a Welch two-sample *t* test (nonpaired). Assumptions of normality were examined graphically.

Statistical significance of the quantification of all phosphorylation sites of proteins in the insulin/TOR signaling pathway (Fig. 6b) was calculated between phosphorylation sites that do (*N* = 87) and do not (*N* = 16,888) belong to the InR/TOR pathway using Welch two-sample *t* test (nonpaired). Assumptions of normality were examined graphically.

GO-term analysis (Fig. 6c) was performed using the FlyMine database^39^ using a Benjamini–Hochberg corrected test.

All *P* values related to peptide phosphorylation sites were calculated using the Limma package in R/Bioconductor^38^ between *N* = 3 well-fed and *N* = 3 protein-deprived samples.

Statistical significance of western blot quantification (Fig. 7c, d) was calculated for *N* = 4 independent experiments using Welch two-sample *t* test (nonpaired).

Statistical significance of GFP-HDAC1 control (*N* = 118 cells from three independent experiments), wt (*N* = 17 cells from three independent experiments), S391A (*N* = 17 cells from three independent experiments), and S391D (*N* = 16 cells from three independent experiments) expression in S2R+ cells (Supplementary Fig. S7a) was calculated using Welch two-sample *t* test (nonpaired). Assumptions of normality were examined graphically.

The significance of changes in kinase consensus motif groups was calculated using the Wilcoxon rank-sum test. Total dataset size was *N* = 16,975 against which the respective kinase consensus site of the indicated kinases (Akt *N* = 24, AMPK N = 16, Aurora *N* = 20, CaMK2 *N* = 162, Cdc2 *N* = 47, CDK1 *N* = 90, CK1 *N* = 167, CK2 *N* = 109, ERK/MAPK *N* = 40, GSK3 *N* = 197, PKA *N* = 351, S6K *N* = 43, TOR *N* = 375) were compared. Assumptions of normality were examined graphically.

### Reporting summary

Further information on research design is available in the Nature Portfolio Reporting Summary linked to this article.

## Supplementary information

Supplementary figures

Description of Additional Supplementary Files

Supplementary Data 1

Supplementary Data 2

Supplementary Data 3

Reporting Summary

## Data Availability

The mass spectrometry phosphoproteomics data have been deposited at the Open Science Framework repository (OSF): https://osf.io/dprt5/?view_only=b3770945c8384fbe962da1518e4f84be. Uncropped/original blots for all figures are available in the Supplementary File as Supplementary Fig. 9. Source data for all graphs are available in Supplementary Data 1. All other data are available from the corresponding author upon reasonable request.

## Code availability

The computer code used for the analysis of the phosphoproteomics data is available from: https://github.com/frankwippich/Wippich_Phosphoproteomics-analysis.

## References

[1] Burn KM (2015). Somatic insulin signaling regulates a germline starvation response in Drosophila egg chambers. Developmental Biol..

[2] Shimada Y, Burn KM, Niwa R, Cooley L (2011). Reversible response of protein localization and microtubule organization to nutrient stress during Drosophila early oogenesis. Dev. Biol..

[3] Parker R, Sheth U (2007). P bodies and the control of mRNA translation and degradation. Mol. Cell.

[4] Janke C, Montagnac G (2017). Causes and consequences of microtubule acetylation. Curr. Biol..

[5] Kalebic N (2013). αTAT1 is the major α-tubulin acetyltransferase in mice. Nat. Commun..

[6] Akella JS (2010). MEC-17 is an α-tubulin acetyltransferase. Nature.

[7] Portran D, Schaedel L, Xu Z, Théry M, Nachury MV (2017). Tubulin acetylation protects long-lived microtubules against mechanical ageing. Nat. Cell Biol..

[8] Reed NA (2006). Microtubule acetylation promotes kinesin-1 binding and transport. Curr. Biol..

[9] Geeraert C (2010). Starvation-induced hyperacetylation of tubulin is required for the stimulation of autophagy by nutrient deprivation. J. Biol. Chem..

[10] Kwon S, Zhang Y, Matthias P (2007). The deacetylase HDAC6 is a novel critical component of stress granules involved in the stress response. Genes Dev..

[11] Hubbert C (2002). HDAC6 is a microtubule-associated deacetylase. Nature.

[12] Matsuyama A (2002). In vivo destabilization of dynamic microtubules by HDAC6-mediated deacetylation. EMBO J..

[13] Zhang Y (2003). HDAC-6 interacts with and deacetylates tubulin and microtubules in vivo. EMBO J..

[14] Segré CV, Chiocca S (2011). Regulating the regulators: the post-translational code of class I HDAC1 and HDAC2. J. Biomed. Biotechnol..

[15] Yang XJ, Seto E (2008). The Rpd3/Hda1 family of lysine deacetylases: from bacteria and yeast to mice and men. Nat. Rev. Mol. Cell Biol..

[16] Khan DH (2013). Protein kinase CK2 regulates the dimerization of histone deacetylase 1 (HDAC1) and HDAC2 during mitosis. J. Biol. Chem..

[17] Bhattacharjee, P. Heidelberg, Univ., Diss. (2012).

[18] Ando R, Mizuno H, Miyawaki A (2004). Regulated fast nucleocytoplasmic shuttling observed by reversible protein highlighting. Science.

[19] Loponte S (2016). Dynamic phosphorylation of histone deacetylase 1 by aurora kinases during mitosis regulates zebrafish embryos development. Sci. Rep..

[20] Pflum MK, Tong JK, Lane WS, Schreiber SL (2001). Histone deacetylase 1 phosphorylation promotes enzymatic activity and complex formation. J. Biol. Chem..

[21] Ustinova K (2020). The disordered N-terminus of HDAC6 is a microtubule-binding domain critical for efficient tubulin deacetylation. J. Biol. Chem..

[22] Zimyanin VL (2008). In vivo imaging of oskar mRNA transport reveals the mechanism of posterior localization. Cell.

[23] Barth JMI, Szabad J, Hafen E, Köhler K (2011). Autophagy in Drosophila ovaries is induced by starvation and is required for oogenesis. Cell Death Differ..

[24] Cho Y, Griswold A, Campbell C, Min KT (2005). Individual histone deacetylases in Drosophila modulate transcription of distinct genes. Genomics.

[25] Tsai SC, Seto E (2002). Regulation of histone deacetylase 2 by protein kinase CK2. J. Biol. Chem..

[26] Bian Y (2013). Global screening of CK2 kinase substrates by an integrated phosphoproteomics workflow. Sci. Rep..

[27] Al Quobaili F, Montenarh M (2012). CK2 and the regulation of the carbohydrate metabolism. Metabolism.

[28] Nouar R (2016). Direct evidence for the interaction of stathmin along the length and the plus end of microtubules in cells. FASEB J..

[29] Stoothoff WH, Johnson GV (2005). Tau phosphorylation: physiological and pathological consequences. Biochim Biophys. Acta.

[30] Sung HH (2008). Drosophila ensconsin promotes productive recruitment of Kinesin-1 to microtubules. Dev. Cell.

[31] Lin M-D, Fan S-J, Hsu W-S, Chou T-B (2006). Drosophila decapping protein 1, dDcp1, is a component of the oskar mRNP complex and directs its posterior localization in the oocyte. Dev. Cell.

[32] Nakamura A, Amikura R, Hanyu K, Kobayashi S (2001). Me31B silences translation of oocyte-localizing RNAs through the formation of cytoplasmic RNP complex during Drosophila oogenesis. Development.

[33] Fan SJ, Marchand V, Ephrussi A (2011). Drosophila Ge-1 promotes P body formation and oskar mRNA localization. PLoS ONE.

[34] Gaspar I, Wippich F, Ephrussi A (2017). Enzymatic production of single-molecule FISH and RNA capture probes. Rna.

[35] Scholz J, Besir H, Strasser C, Suppmann S (2013). A new method to customize protein expression vectors for fast, efficient and background free parallel cloning. BMC Biotechnol..

[36] Hennrich ML (2018). Cell-specific proteome analyses of human bone marrow reveal molecular features of age-dependent functional decline. Nat. Commun..

[37] Huber W, von Heydebreck A, Sültmann H, Poustka A, Vingron M (2002). Variance stabilization applied to microarray data calibration and to the quantification of differential expression. Bioinformatics.

[38] Ritchie ME (2015). limma powers differential expression analyses for RNA-sequencing and microarray studies. Nucleic Acids Res..

[39] Lyne R (2007). FlyMine: an integrated database for Drosophila and Anopheles genomics. Genome Biol..

[40] Shida T, Cueva JG, Xu Z, Goodman MB, Nachury MV (2010). The major α-tubulin K40 acetyltransferase αTAT1 promotes rapid ciliogenesis and efficient mechanosensation. Proc. Natl Acad. Sci. USA.

