# Supplementary material for: Nutritional stress-induced regulation of microtubule organization and mRNP transport by HDAC1 controlled α-tubulin acetylation
Source: Commun Biol. 2023 Jul 25;6:776. doi: 10.1038/s42003-023-05138-w (PMC10368696; doi:10.1038/s42003-023-05138-w)
Supplement: Supplementary file 1 — Supplementary figures [file 42003_2023_5138_MOESM1_ESM.pdf]

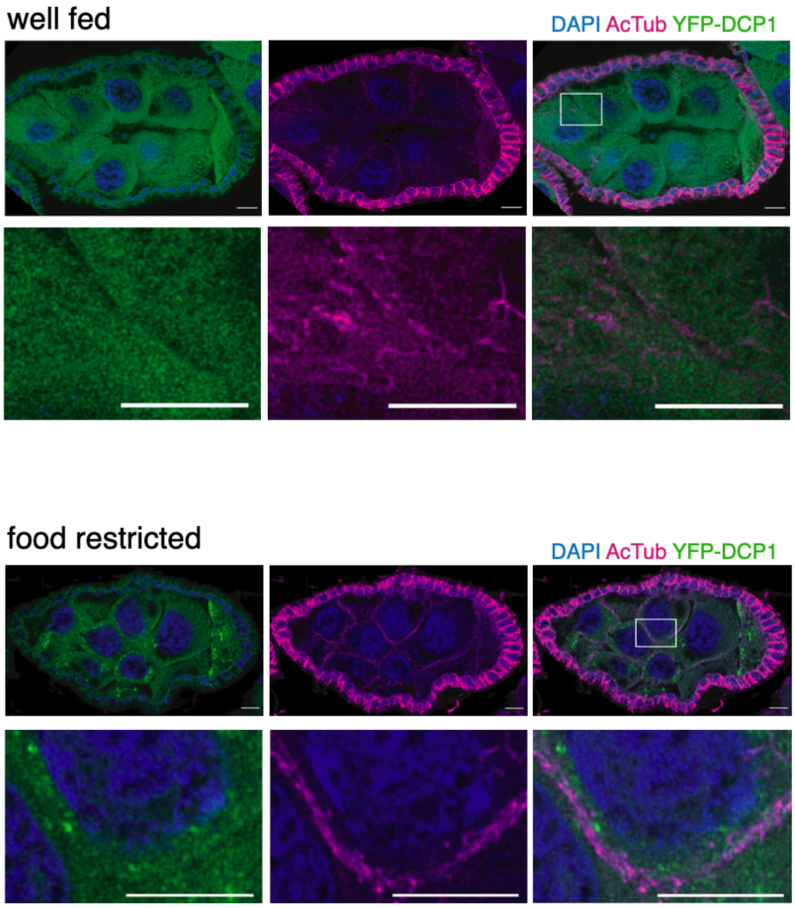

Supplementary Figure 1: Starvation induces P-body formation and cortical accumulation of acetylated microtubules. P-bodies are visualized by staining for YFP-DCP1, a known P-body marker (Lin *et al.*, 2006). The lower panels show magnified images of a cell boundary within the rectangular area highlighted in the upper panels on the right (merged signals). Scale bar: 10μm

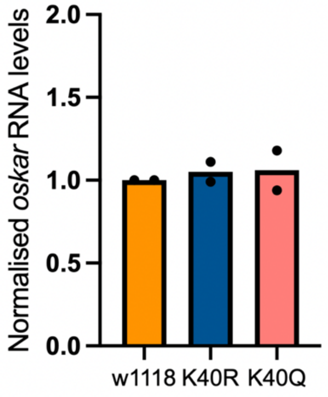

Supplementary Figure 2: *oskar* mRNA levels are unaffected in K40R, K40Q expressing flies compared with the w1118 control. *oskar* mRNA levels were quantified by qPCR and normalized to total 18S rRNA levels.

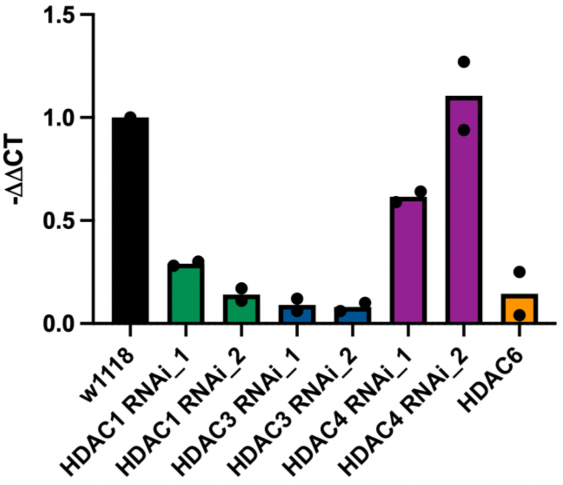

Supplementary Figure 3: RNAi knock-down of HDAC1 (RNAi 1 and 2), HDAC3 (RNAi 1 and 2), and HDAC6 was efficient, as determined by qPCR. Knock-down of HDAC4 by RNAi 1 was less efficient (~50%), whereas RNAi 2 had no effect. 18S rRNA levels were used for normalization.

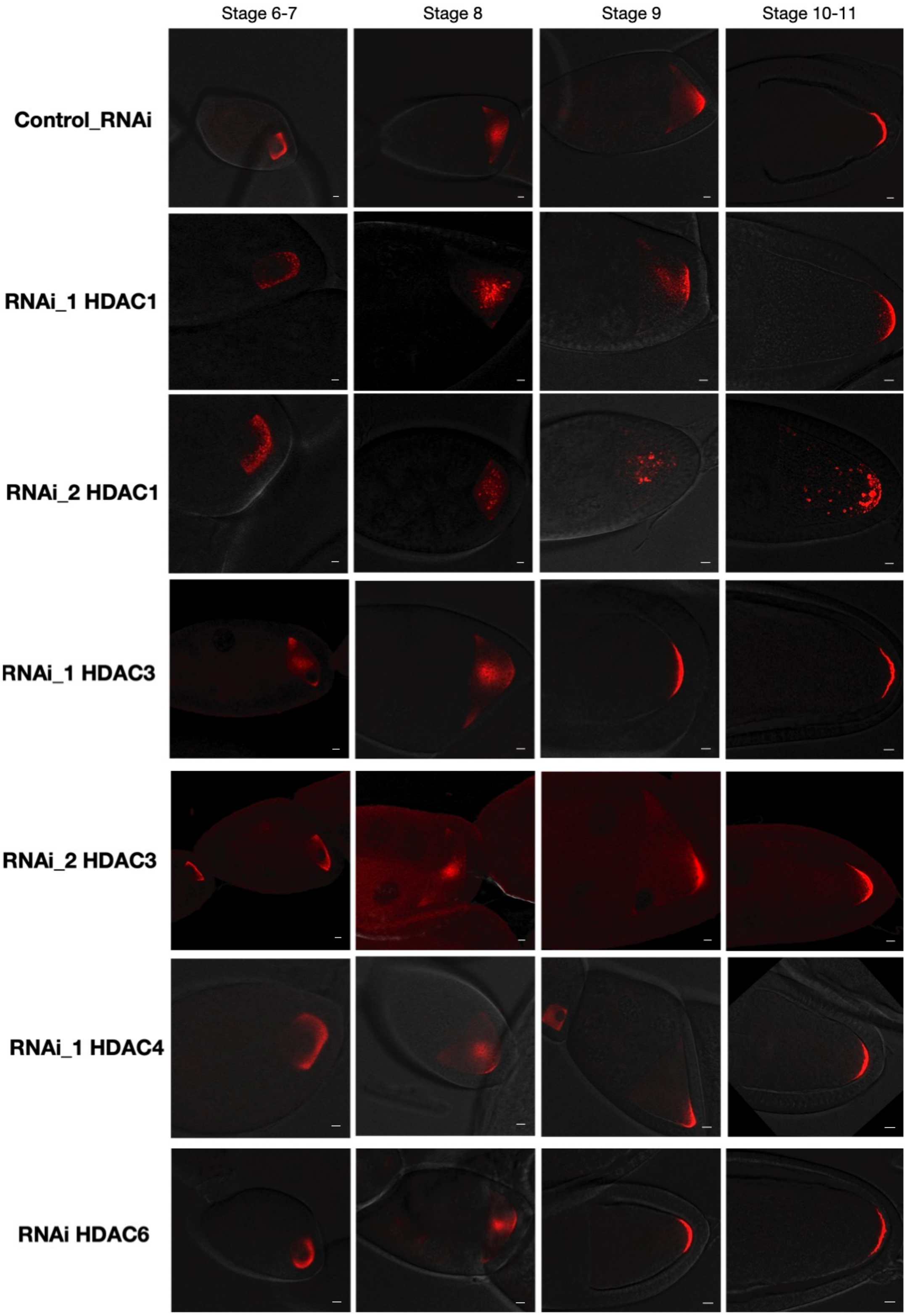

Supplementary Figure 4: Knock-down of HDAC1, but not other histone deacetylases, leads to mRNA aggregation in well-fed flies. *oskar* mRNA detected by smFISH. Panels from left to right show oocytes at progressive stages of development. Scale bars: 5μm

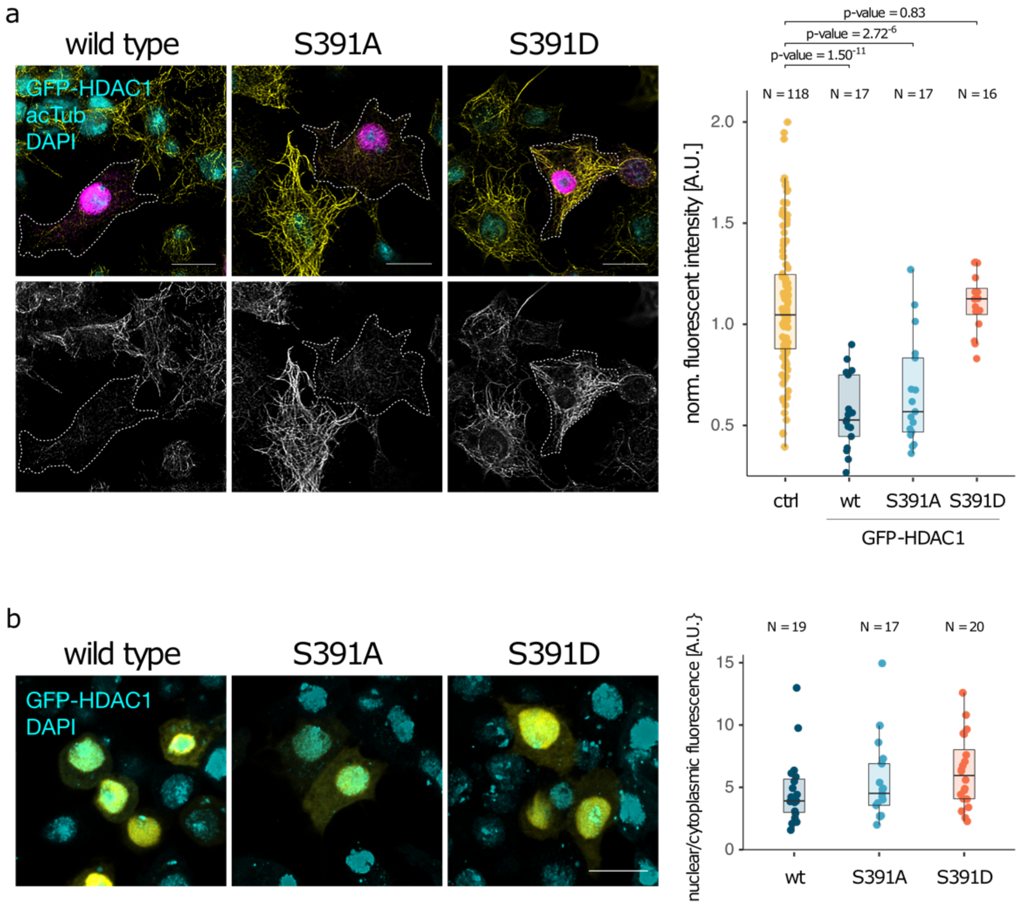

Supplementary Figure 5: HDAC1 wild type and mutant overexpression in S2R+ cells. (a) S2R+ cells transiently transfected with GFP-HDAC1 wild type, S391A or S391D stained 24 hours post transfection for acetylated α-tubulin K40 and DAPI. The fluorescence intensity of acetylated α-tubulin K40 was quantified. Non transfected cells served as control. Statistical significance was calculated using Welch Two Sample t-test. (b) The nucleocytoplasmic ratio of S2R+ cells overexpressing GFP-HDAC1 wild type, S391A or S391D was quantified. Scale bars: 10 µm.

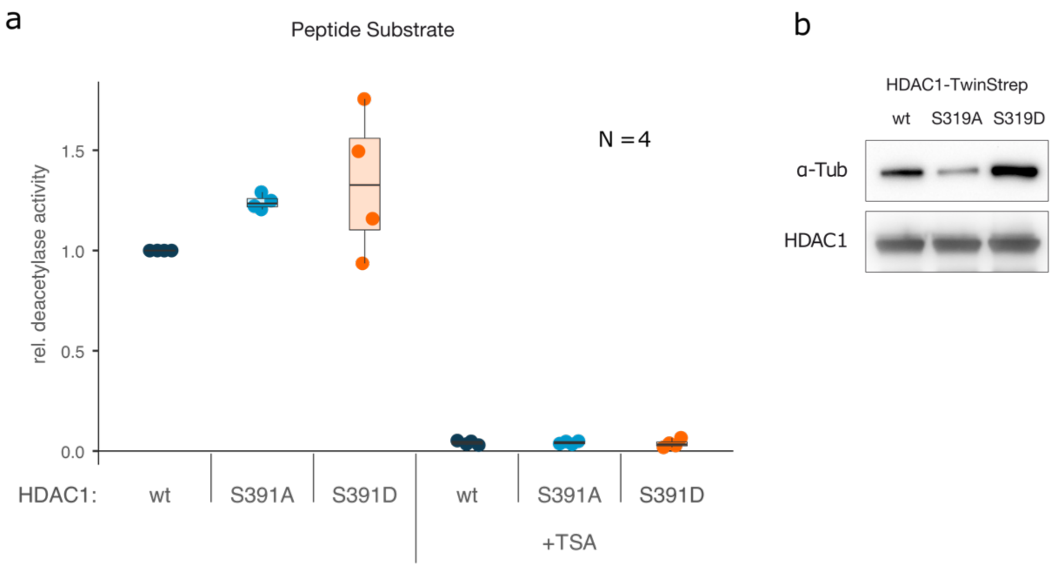

Supplementary Figure 6: The effect of HDAC1 S319 mutations on the deacetylation activity of short peptide substrates and α-tubulin binding. (a) Recombinant HDAC1 wild type, S391A or S391D was used in a fluorimetric activity assay using short peptide substrates. The error bars represent standard deviation. (b) Western blot analysis of α-tubulin copurified with HDAC1-TwinStrep wild type, S391A or S391D expressed in insect cells.

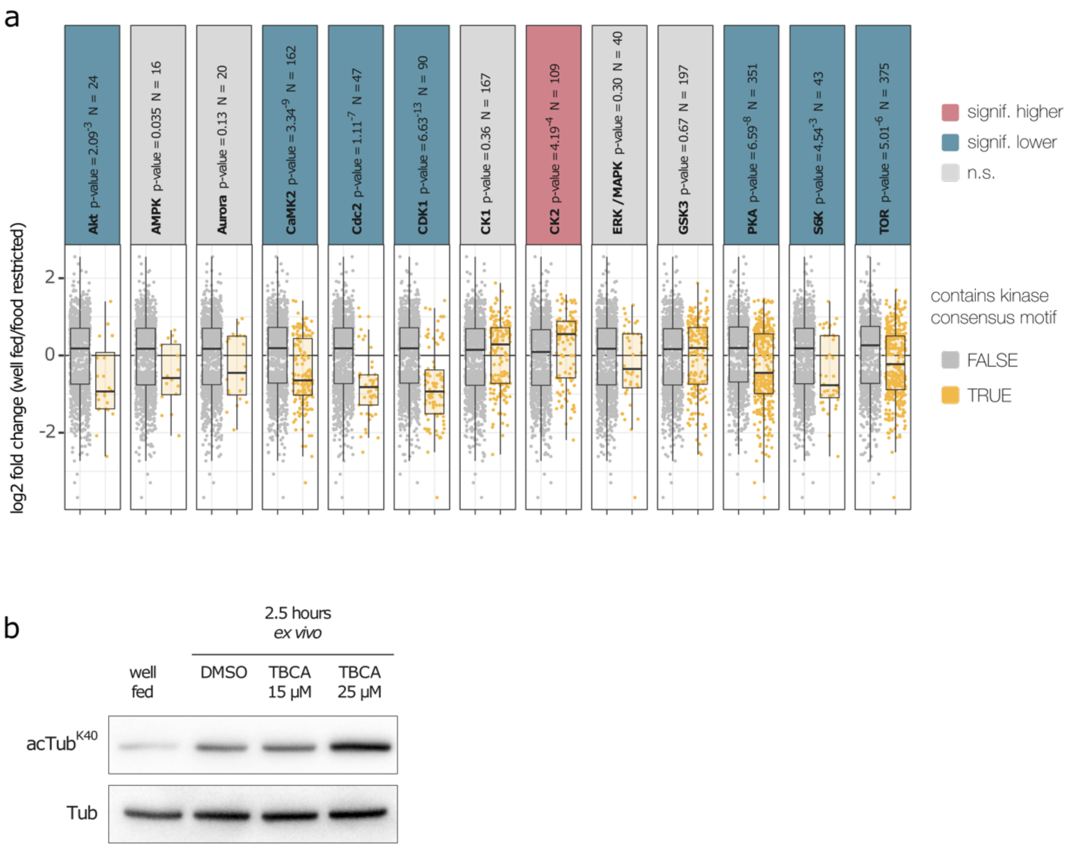

Supplementary Figure 7: Kinases involved in nutritional stress transmission. (a) Analysis of the response of phosphorylation sites belonging to the indicated kinase consensus motif group to nutritional stress. Kinase consensus motif groups that change significantly are highlighted (blue: less phosphorylated during nutritional stress; red: more phosphorylated during nutritional stress). Statistical significance was calculated using Wilcoxon rank sum test. (b) Acetylation of α-tubulin in ovaries from well-fed females or *ex vivo* cultured in the presence of DMSO control or TBCA was analysed by western blot.

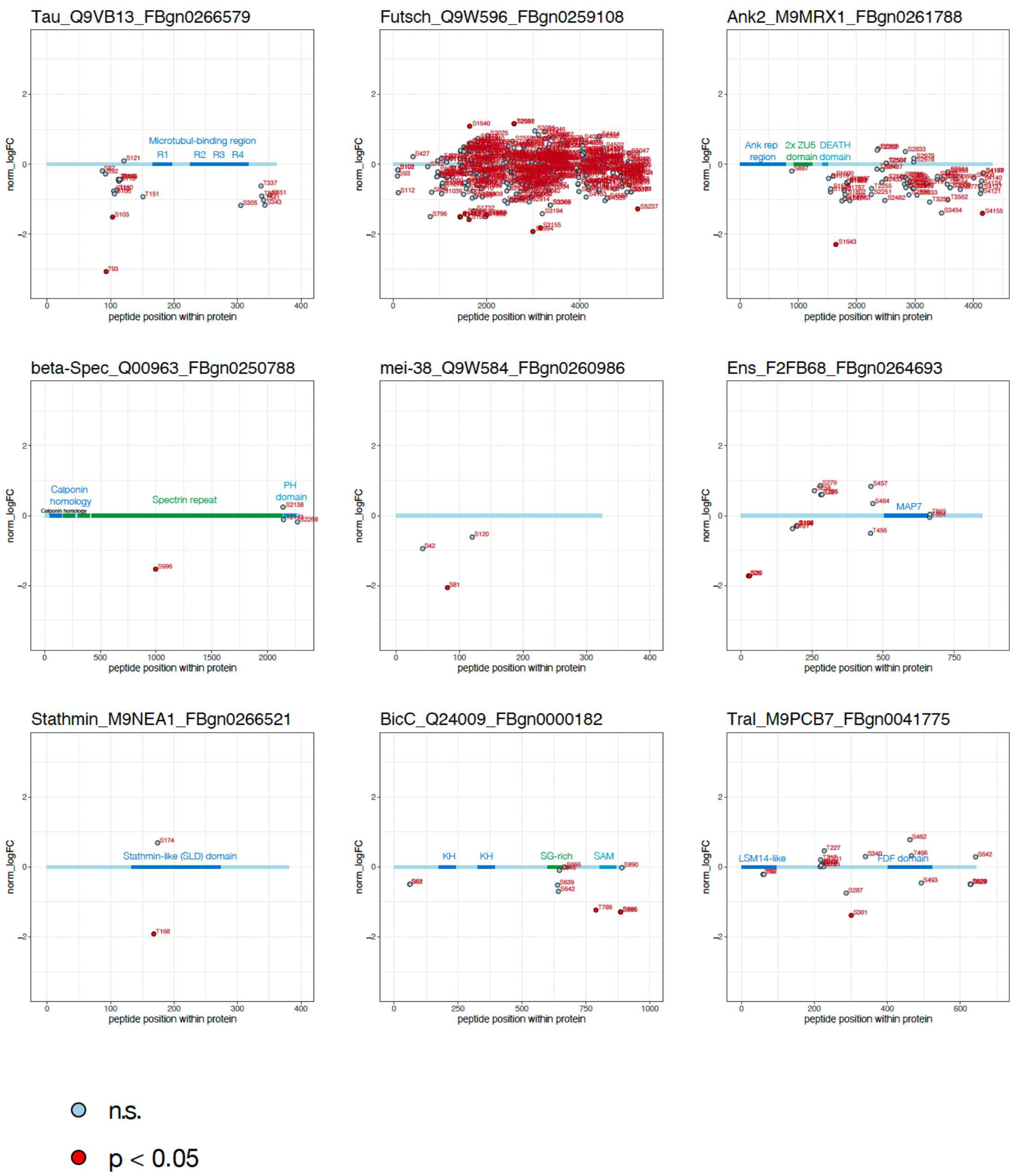

Supplementary Figure 8: Examples of microtubule associated proteins whose phosphorylation status was significantly altered in response to nutritional stress. Visualization of example proteins (Name_UniProtID_FlybaseID), domains and the phosphorylation sites identified by quantitative phosphoproteomics.

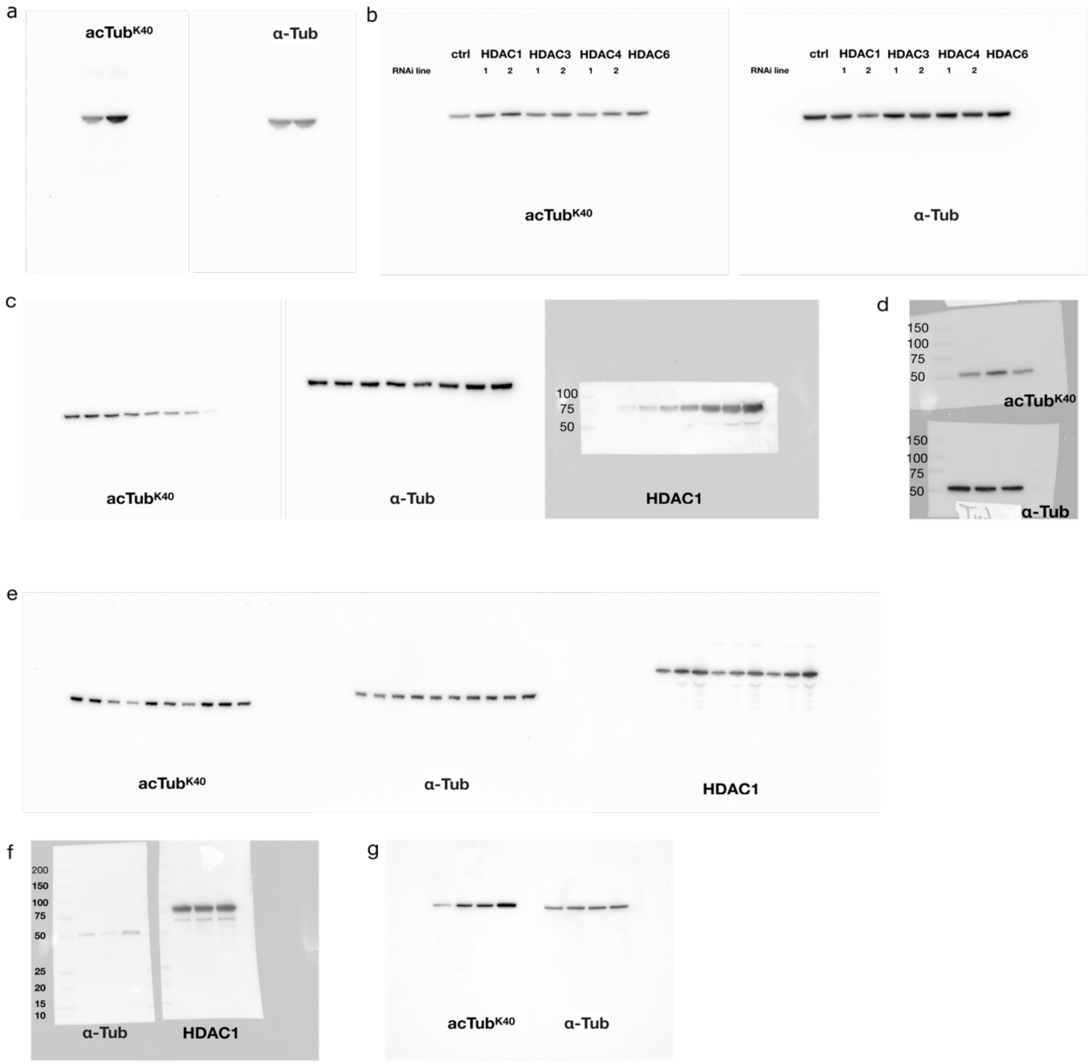

Supplementary Figure 9: Uncropped/original blots shown in the manuscript. (a) Blot of Figure 1c. (b) Blot of Figure 3b (Note that HDAC4 RNAi_2 is not included in the study due to ineffective knockdown). (c) Blot of Figure 4a. (d) Blot of Figure 5c. (e) Blot of Figure 7c. (f) Blot of Supplementary Figure S6b. (g) Blot of Supplementary Figure S7b.
