# Supplementary material for: Nutritional stress-induced regulation of microtubule organization and mRNP transport by HDAC1 controlled α-tubulin acetylation
Source: Commun Biol. 2023 Jul 25;6:776. doi: 10.1038/s42003-023-05138-w (PMC10368696; doi:10.1038/s42003-023-05138-w)
Supplement: Supplementary file 2 — Description of Additional Supplementary Files [file 42003_2023_5138_MOESM2_ESM.docx]

**Description of Additional Supplementary Files**

**File name:** Supplementary Data 1

**Description:** The source data behind graphs in Figures 1C, 5B, 7D, and Supplementary Figures S2, S3, S5A, S5B, and S6A.

**File name:** Supplementary Data 2

**Description:** Results of the phosphoproteomics analysis of egg-chambers from well-fed and nutrient deprived conditions

**File name:** Supplementary Data 3

**Description:** Gene ontology (GO) term analysis of proteins whose phosphorylation sites were significantly downregulated (p-value < 0.05)
