# Supplementary material for: Nutritional stress-induced regulation of microtubule organization and mRNP transport by HDAC1 controlled α-tubulin acetylation
Source: Commun Biol. 2023 Jul 25;6:776. doi: 10.1038/s42003-023-05138-w (PMC10368696; doi:10.1038/s42003-023-05138-w)
Supplement: Supplementary file 6 — Reporting Summary [file 42003_2023_5138_MOESM6_ESM.pdf]

nature portfolio

Corresponding author(s): Anne Ephrussi

Last updated by author(s): May 19, 2023

Reporting Summary

Nature Portfolio wishes to improve the reproducibility of the work that we publish. This form provides structure for consistency and transparency in reporting. For further information on Nature Portfolio policies, see our Editorial Policies and the Editorial Policy Checklist.

Statistics

For all statistical analyses, confirm that the following items are present in the figure legend, table legend, main text, or Methods section.

n/a	Confirmed	
☐	☒	The exact sample size (*n*) for each experimental group/condition, given as a discrete number and unit of measurement
☒	☐	A statement on whether measurements were taken from distinct samples or whether the same sample was measured repeatedly
☐	☒	The statistical test(s) used AND whether they are one- or two-sided<br>*Only common tests should be described solely by name; describe more complex techniques in the Methods section.*
☐	☒	A description of all covariates tested
☒	☐	A description of any assumptions or corrections, such as tests of normality and adjustment for multiple comparisons
☐	☒	A full description of the statistical parameters including central tendency (e.g. means) or other basic estimates (e.g. regression coefficient) AND variation (e.g. standard deviation) or associated estimates of uncertainty (e.g. confidence intervals)
☒	☐	For null hypothesis testing, the test statistic (e.g. *F*, *t*, *r*) with confidence intervals, effect sizes, degrees of freedom and *P* value noted<br>*Give P values as exact values whenever suitable.*
☒	☐	For Bayesian analysis, information on the choice of priors and Markov chain Monte Carlo settings
☒	☐	For hierarchical and complex designs, identification of the appropriate level for tests and full reporting of outcomes
☒	☐	Estimates of effect sizes (e.g. Cohen's *d*, Pearson's *r*), indicating how they were calculated

Our web collection on statistics for biologists contains articles on many of the points above.

Software and code

Policy information about availability of computer code

Data collection	NA
Data analysis	Thermo Proteome Discoverer version 1.4.1.14<br>Percolator version 2.04<br>PhosphorRS 3.0<br>The custom computer code used for further analysis of the phosphoproteomics data is available from https://github.com/frankwippich/Wippich_Phosphoproteomics-analysis

For manuscripts utilizing custom algorithms or software that are central to the research but not yet described in published literature, software must be made available to editors and reviewers. We strongly encourage code deposition in a community repository (e.g. GitHub). See the Nature Portfolio guidelines for submitting code & software for further information.

Data

Policy information about availability of data

All manuscripts must include a data availability statement. This statement should provide the following information, where applicable:

- Accession codes, unique identifiers, or web links for publicly available datasets
- A description of any restrictions on data availability
- For clinical datasets or third party data, please ensure that the statement adheres to our policy

The mass spectrometry phosphoproteomics data have been deposited at the Open Science Framework repository (OSF).

Human research participants

Policy information about studies involving human research participants and Sex and Gender in Research.

Reporting on sex and gender	na
Population characteristics	na
Recruitment	na
Ethics oversight	na

Note that full information on the approval of the study protocol must also be provided in the manuscript.

Field-specific reporting

Please select the one below that is the best fit for your research. If you are not sure, read the appropriate sections before making your selection.

☒ Life sciences ☐ Behavioural & social sciences ☐ Ecological, evolutionary & environmental sciences

For a reference copy of the document with all sections, see nature.com/documents/nr-reporting-summary-flat.pdf

Life sciences study design

All studies must disclose on these points even when the disclosure is negative.

Sample size	Sample size is indicated for individual experiments
Data exclusions	na
Replication	All replications included in the data
Randomization	na
Blinding	na

Reporting for specific materials, systems and methods

We require information from authors about some types of materials, experimental systems and methods used in many studies. Here, indicate whether each material, system or method listed is relevant to your study. If you are not sure if a list item applies to your research, read the appropriate section before selecting a response.

Materials & experimental systems

n/a	Involved in the study
☐	☒ Antibodies
☐	☒ Eukaryotic cell lines
☒	☐ Palaeontology and archaeology
☐	☒ Animals and other organisms
☒	☐ Clinical data
☒	☐ Dual use research of concern

Methods

n/a	Involved in the study
☒	☐ ChIP-seq
☒	☐ Flow cytometry
☒	☐ MRI-based neuroimaging

Antibodies

Antibodies used	Mouse anti-α-Tubulin (Sigma, T5168), mouse anti-acetylated-α-Tubulin (Sigma, T7451), mouse anti-Me31B, rat anti-dGe1 and rabbit anti-HDAC1 (Jürg Müller).
Validation	Sigma, T5168:<br>Validated by manufacturer. Recognizes an epitope located at the C-terminal end of the α-tubulin isoform in a variety of organisms.<br>Sigma, T7451:<br>Validated by manufacturer. The antibody has been used to detect acetylated α-tubulins from many organisms including protista, plants, invertebrates, and vertebrates.<br>mouse anti-Me31B:<br>Validated by IF in me31B null tissue sample (see Nakamura, A., Amikura, R., Hanyu, K. & Kobayashi, S. Me31B silences translation of oocyte-localizing RNAs through the formation of cytoplasmic RNP complex during Drosophila oogenesis. Development 128, 3233–3242 (2001).).<br>rat anti-dGe1:<br>Validated by WB in dGe1-Δ5 mutant flies (see Fan, S.-J., Marchand, V. & Ephrussi, A. Drosophila Ge-1 promotes P body formation and oskar mRNA localization. PLoS ONE 6, e20612 (2011).).<br>rabbit anti-HDAC1:<br>Validated by recombinant HDAC1 in Figure 3.

Eukaryotic cell lines

Policy information about cell lines and Sex and Gender in Research

Cell line source(s)	S2R+ from Drosophila Genomics Resource Center, Stock Number 150
Authentication	none of the cell lines were authenticated
Mycoplasma contamination	the cell lines were not tested for Mycoplasma contamination
Commonly misidentified lines (See ICLAC register)	*Name any commonly misidentified cell lines used in the study and provide a rationale for their use.*

Animals and other research organisms

Policy information about studies involving animals; ARRIVE guidelines recommended for reporting animal research, and Sex and Gender in Research

Laboratory animals	Drosophila melanogaster:<br>OregonR, w1118 was used as wild type. OregonR (Bloomington Stock 5#) was used for mass extraction of ovaries for phosphoproteomics. RNAi against HDAC1 (RNAi_1: Bloomington Stock #36800; RNAi_2: Bloomington Stock #33725), HDAC3 (RNAi_1: Bloomington Stock #64476; RNAi_2: Bloomington Stock #34778), HDAC4 (RNAi_1: Bloomington Stock #34774; RNAi_2: Bloomington Stock #28549), HDAC6 (RNAi_1: Bloomington Stock #34072) and control RNAi (Bloomington Stock #35573) were driven by MTD-Gal4 (Bloomington Stock # 31777).
Wild animals	*Provide details on animals observed in or captured in the field; report species and age where possible. Describe how animals were caught and transported and what happened to captive animals after the study (if killed, explain why and describe method; if released, say where and when) OR state that the study did not involve wild animals.*
Reporting on sex	*Indicate if findings apply to only one sex; describe whether sex was considered in study design, methods used for assigning sex. Provide data disaggregated for sex where this information has been collected in the source data as appropriate; provide overall numbers in this Reporting Summary. Please state if this information has not been collected. Report sex-based analyses where performed, justify reasons for lack of sex-based analysis.*
Field-collected samples	*For laboratory work with field-collected samples, describe all relevant parameters such as housing, maintenance, temperature, photoperiod and end-of-experiment protocol OR state that the study did not involve samples collected from the field.*
Ethics oversight	*Identify the organization(s) that approved or provided guidance on the study protocol, OR state that no ethical approval or guidance was required and explain why not.*

Note that full information on the approval of the study protocol must also be provided in the manuscript.
